# Niche Differentiation and Predicted Functions of Microbiomes in a Tri-Trophic Willow–Gall (*Euura viminalis*)–Parasitoid Wasp System

**DOI:** 10.3390/insects17010043

**Published:** 2025-12-29

**Authors:** Yuhao Nie, Gaopeng Yu, Hongying Hu

**Affiliations:** College of Life Science and Technology, Xinjiang University, Urumqi 830017, China

**Keywords:** parasitoid, gall wasp, willow, symbiotic microbiota, high-throughput sequencing, niche differentiation

## Abstract

Chalcidoids (Hymenoptera: Chalcidoidea), the most important natural enemies of parasitoids, serve as a pivotal factor in the regulation and management of pest populations. Microbiotas mediate interactions among plants, herbivores, and natural enemies and shape host immunity, parasitoid development, and gall formation; however, the niche-specific diversity and functions of tritrophic parasitoid–host–gall systems remain unclear. Focusing on leaf galls induced on twisted willow (*Salix matsudana* f. *tortuosa*) by the willow-galling sawfly *Euura viminalis* and on two chalcidoids, *Eurytoma aethiops* and *Aprostocetus* sp., we profiled bacterial and fungal microbiomes across plant surfaces, gall lumen, host larval tissues, and parasitoids using HTAS. Fungal diversity peaked on parasitoids but was depleted in the gall lumen and host tissues; bacterial richness showed the opposite trend, peaking in the gall lumen and decreasing on parasitoids. In networks contrasted by kingdom, fungi showed positive interface-hub connectivity (*Cladosporium*, *Alternaria*), whereas bacteria showed negative hub-mediated associations (*Pseudomonas*, *Acinetobacter*), indicating habitat-specific replacements: exposed niches favored transport, two-component, secretion–motility and energy functions, whereas the gall lumen reduced transport/motility but selectively retained N/S metabolism; and in host tissues, information processing and nitrogen respiration were highlighted. These results inform microbiome-guided parasitoid biocontrol.

## Simple Summary

Symbiotic microbes support insect survival and development, yet how niches shape them in a willow–gall–parasitoid system is unclear. We sampled six types of sites—leaf and gall surfaces, gall interiors, sawfly larvae, and wasps—and used genetic sequencing to identify bacteria and fungi. A clear pattern emerged: open, air-exposed sites (leaf and gall surfaces and wasps) held richer, more balanced fungal communities, whereas the sealed gall interiors and larvae contained few fungi. Bacteria showed the opposite pattern, with the highest diversity inside galls. Habitats organized these communities more than host identity, informing more precise, microbe-aware pest control programs.

## 1. Introduction

The superfamily Chalcidoidea (Hymenoptera) represents one of the most diverse and ecologically significant groups of parasitoid wasps, comprising more than 22,500 described species worldwide, with total diversity potentially exceeding half a million species in [1,2,3]. Chalcidoid parasitoids exhibit remarkable host specificity and niche differentiation and act as key natural enemies that regulate herbivorous insect populations [4,5,6]. They furnish essential ecosystem services that enhance long-term pest control and ecological stability [7,8,9] because they are commonly utilized for pest management in agricultural production systems [10,11,12,13].

**Figure 1:**
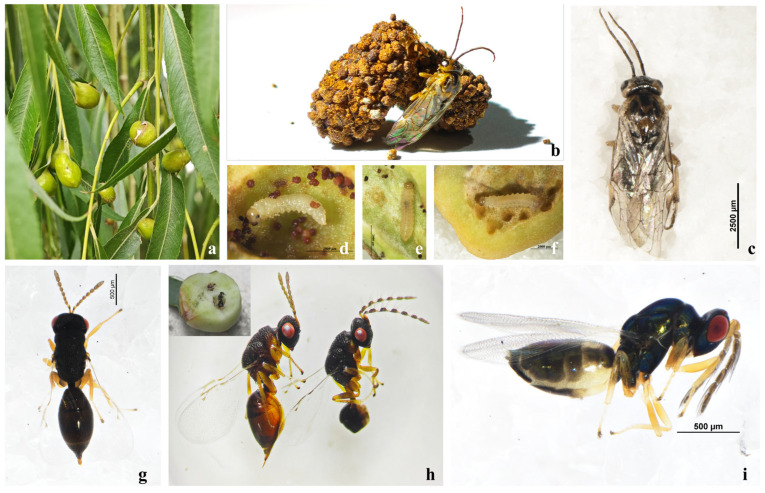
Study system and focal insects of the willow–gall–parasitoid complex. (**a**) Ecological view of galls; (**b**,**c**) adults of *Euura viminalis*; (**d**–**f**) *E. viminalis* larvae inside galls; (**g**,**h**) *Eurytoma aethiops* female (left) and a male (right); (**i**) *Aprostocetus* sp.

**Figure 2:**
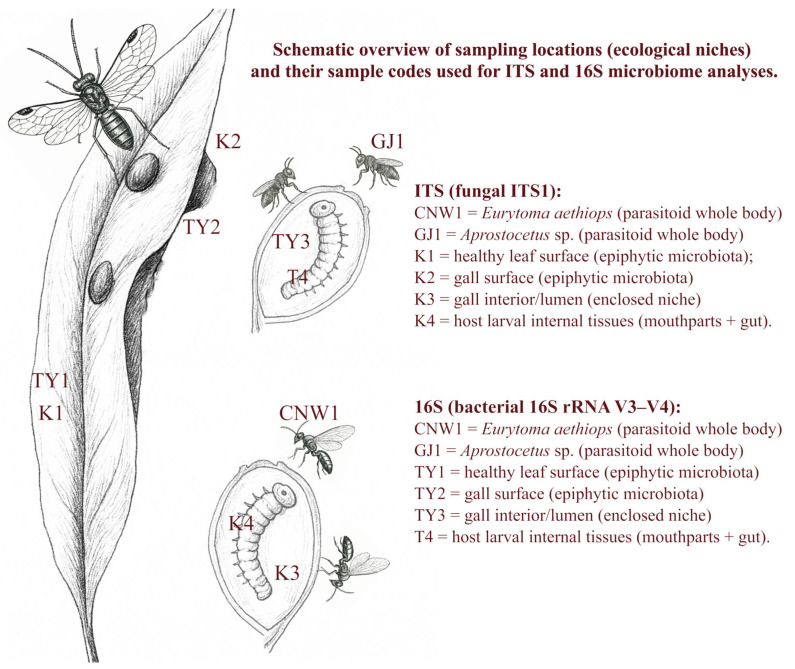
Conceptual schematic (not to scale) of sampling locations (ecological niches) and their sample codes used for ITS and 16S microbiome analyses.

**Figure 3:**
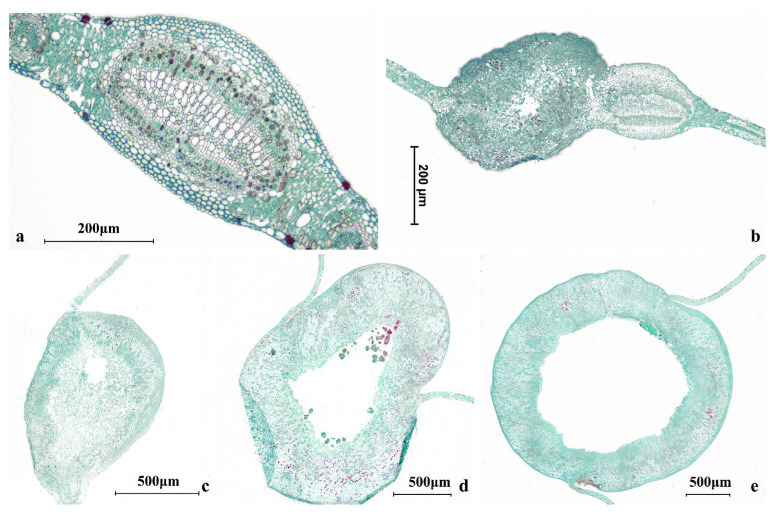
Anatomical sections of willow leaf galls. (**a**–**e**) Progressive enlargement accompanies larval growth in *E. viminalis*. (**a**) Initiation young stage; (**b**) young stage; (**c**) developing stage; (**d**) expanding stage; (**e**) mature stage.

The willow-galling sawfly *Euura viminalis* (Linnaeus, 1758) induces globose galls on the leaves of willow, with each gall typically containing a single larva [14,15]. Each leaf usually has 1~4 galls (Figure 1 and Figure 2), and these structures impose a heavy metabolic burden on the tree, inducing leaf malformation, reducing photosynthesis, and weakening overall vigor [16,17]. Within each gall, the larva develops in a distinct internal chamber surrounded by a thickened gall wall; representative cross-sections (Figure 3) illustrate this basic architecture and show that tissues bordering the larval chamber appear relatively compact, with smaller and more tightly packed cells than surrounding tissues, providing structural context for defining the sampling compartments used in our niche-based microbiome analyses. When populations of *E. viminalis* undergo outbreaks, infestations can significantly damage the ornamental and ecological functions of willows, including their roles in wind prevention and sand fixation [18,19]. Consequently, the *E. viminalis*–parasitoid–willow system represents an important model for studying multitrophic plant–insect interactions and serves as an ecologically relevant framework for forest protection and landscape management [20,21,22].

In recent years, research on insect microbiomes has revealed that microbial symbionts play crucial roles in mediating ecological interactions across multiple trophic levels [23,24]. Microbial communities regulate host physiology and behavior through nutrient metabolism, detoxification, immune modulation, and stress tolerance [25,26]. In parasitoid wasps, microbial symbionts—including bacteria, fungi, and mutualistic viruses—contribute to host manipulation and immune suppression, thereby facilitating successful parasitism and offspring development [23,24]. Galls induced by herbivorous insects provide enclosed, resource-rich microhabitats that foster diverse microbial assemblages, which can in turn influence both gall formation and the outcomes of parasitism [27,28]. Moreover, parasitoid-mediated changes in the herbivore microbiota can cascade to affect plant defense signaling and resource allocation [25]. Collectively, the relevant studies highlight the microbiome as an often overlooked but central mediator within plant–herbivore–parasitoid tritrophic systems, shaping ecological dynamics and potentially influencing biological control outcomes [26,29].

Despite these advances, research on microbial communities in parasitoid–host–gall multitrophic systems remains comparatively limited in scope, particularly regarding niche differentiation and ecological functions [30,31]. Previous investigations have focused primarily on plant–herbivore interactions or on the rhizosphere microbiota associated with soil animals, while the bacterial and fungal communities inhabiting distinct ecological microhabitats, such as parasitoid wasps, gall surfaces, gall interiors, and host larvae, have received limited attention [32,33,34]. Recent studies have shown that galling behavior and host specialization are closely associated with symbiont composition and metabolic capabilities, suggesting that microhabitat structure may drive microbial community divergence [31,35,36]. Functional prediction and metagenomic approaches, including PICRUSt- and KEGG-based analyses, have revealed that metabolic and nutrient-cycling pathways are often enriched in galling or parasitic insects, but the empirical validation of these inferred functions remains limited [37,38,39]. Moreover, microbiome differentiation along ecological gradients has been documented in various insects and host-associated environments, reinforcing the notion that both host traits and environmental context shape microbial assembly and function [40,41]. Although alpha- and beta-diversity metrics provide an overview of which microbial taxa are present, functional prediction remains crucial for understanding how these microorganisms contribute to nutrient cycling, metabolic adaptation, detoxification, and the regulation of host–parasitoid interactions [31,34,42,43]. These insights underscore the urgent need for integrative multiomics and ecological network approaches to disentangle the functional significance of microbial communities within parasitoid–host–gall systems.

This research focused on willow leaves, *E. viminalis* larvae and galls, and the parasitoid wasps *E. aethiops* and *Aprostocetus* sp. This three-trophic-level system is taken as the study object. By high-throughput sequencing of the 16S rRNA gene and ITS regions, the bacterial and fungal microbiomes were characterized across six ecological niches (individual parasitoid wasps, healthy leaf surfaces, gall surfaces, gall interiors, and internal tissues of gall inducers), following standard amplicon-based microbial ecology approaches [44,45]. Community diversity analysis and functional prediction were performed using Tax4Fun2, FAPROTAX, and FUNGuild, which have been widely applied in ecological and entomological microbiome studies to predict metabolic and ecological functions on the basis of marker–gene datasets [46,47,48]. Functional annotations and redundancy analyses were combined with diversity indices to clarify the microbial composition and niche differentiation [38,40]. These methods have been successfully used elsewhere to investigate multitrophic microbial interactions and host adaptation in gall-inducing and parasitoid insect systems [28,35]. This integrated approach provides robust microbiological evidence for understanding the ecological adaptation of parasitoid wasps and offers new insights into microbiome-based biological control strategies [24].

## 2. Materials and Methods

### 2.1. Sample Collection

Leaf galls induced on willow by *E. viminalis* were sampled in Bosten Lake National Wetland Park, Bohu County, Bayingolin Mongolian Autonomous Prefecture, Xinjiang, China (approx. 41.90° N, 86.70° E; ~1050 m a.s.l.) on 15 September 2023, which coincided with peak galling and parasitoid activity. Three transects (>200 m apart) were established; within each transect, one biological replicate per niche was collected at a randomized position ≥30 m from other collection sites. The sampling order was computer randomized, and labeling and DNA extraction were performed by the same operator, who was blinded to group identity [49]. We defined six ecological niches for microbiome profiling in this tri-trophic willow–gall–parasitoid system: (1) healthy leaf surface, (2) gall surface, (3) gall lumen/interior, (4) host larval internal tissues, (5) parasitoid body of *Eurytoma aethiops*, and (6) parasitoid body of *Aprostocetus* sp. The leaf surface and gall surface represent exposed epiphytic niches and were sampled by collecting surface-associated microbial material without surface sterilization. The gall lumen/interior represents an enclosed niche and was collected after disinfecting the gall surface and aseptically opening the gall to obtain lumen material and inner-wall biofilm. *E. viminalis* larval internal tissues represent an enclosed internal niche and were collected after larval surface disinfection followed by aseptic dissection of internal tissues (mouthparts + gut). Parasitoid microbiomes were obtained from pooled adult individuals processed as whole bodies. Niche codes used in the ITS dataset include CNW1, GJ1, K1–K4, and those used in the 16S dataset include CNW1, GJ1, TY1–TY3, and T4 (Figure 2). Each niche comprised three independent biological replicates (*n* = 3). Plant-derived replicates were taken from different plants/galls; parasitoid replicates consisted of within-replicate pools (median 15 individuals; range 10–20) and were treated as the experimental unit. Leaf and gall surfaces were processed as epiphytic microbiota without surface sterilization. For gall-interior niches (K3/TY3), galls were surface-sterilized (70% ethanol, 30 s; 1% NaClO, 60 s; three sterile-water rinses) and opened aseptically, after which the larvae of *E. viminalis* were removed. Lumen contents together with inner-wall biofilms were collected by sterile scraping and swabbing, according to aseptic techniques used in insect microbiome studies [50]. In the host, larvae were surface-sterilized as described above and dissected under a stereomicroscope, and their mouthparts and gut were isolated and pooled for each replicate, following procedures adapted for insect gut microbial community profiling [51]. Parasitoid wasps were processed as whole individuals and cryopulverized in liquid nitrogen without prior surface sterilization to reflect the intact parasitoid-associated microbiome. This method captures both internal and surface-associated symbionts and has been successfully applied to hymenopteran parasitoids [52,53]. All procedures involved the use of sterile gloves and instruments to minimize contamination; samples were flash-frozen and stored at −80 °C to preserve microbial DNA integrity [54]. Because larvae and gall interiors occupy enclosed microhabitats, our surface sterilization and aseptic handling are expected to minimize background contamination; given the small size and dissection constraints of parasitoid wasps, we processed whole individuals to comprehensively profile the symbiotic microbiota.

### 2.2. Histological Preparation of Gall Sections

Representative galls spanning the size range encountered in this study were prepared to provide anatomical reference for visualizing the sampling compartments used in the niche-based microbiome analyses. To cover typical field variability, galls were categorized into three size classes (small, medium, and large; corresponding to Figure 3a–c) based on external size, and representative individuals were selected for sectioning.

Galls were fixed, dehydrated through a graded ethanol series, cleared, paraffin-embedded, and serially sectioned at 8–10 μm using a rotary microtome. Sections were mounted on glass slides, deparaffinized, rehydrated, stained with Safranin O–Fast Green, and permanently mounted following routine histological procedures. Images were acquired under bright-field microscopy, and scale bars were added based on microscope calibration. For orientation/background, Figure 3a–e were described as five morphology-based relative stages: (a) initiation young stage (no distinct large chamber or chamber not clearly defined), (b) young stage (incipient chamber features become apparent), (c) developing stage (a more clearly defined internal space with a thickening gall wall), (d) expanding stage (markedly enlarged chamber and thicker wall), and (e) mature stage (stable structure with a thick wall surrounding a large chamber).

These cross-sections are included for orientation/background only and were not used for quantitative anatomical comparisons or for physiological/histochemical interpretation.

### 2.3. DNA Extraction and Sequencing

Genomic DNA was extracted with the FastDNA SPIN Kit for Soil (MP Biomedicals) following the manufacturer’s protocol from ~0.25 g of homogenate (or the entire parasitoid pool for each replicate), as is commonly performed in insect microbiome studies [55,56]. DNA integrity was assessed on 1% agarose gels, and the concentration/purity was measured with a NanoDrop 2000 (A260/280 = 1.8–2.0). The ITS1 region was amplified with the primers ITS1F/ITS2R (~310 bp) for fungi and the bacterial 16S rRNA V3–V4 region with the primers 338F/806R (~460 bp), which have been widely used for microbial community profiling [57,58]. PCR (25 µL) contained 12.5 µL of 2× Phusion High-Fidelity Master Mix, 0.5 µM of each primer, and ~10 ng of template; the cycling conditions were 98 °C for 30 s; 30 cycles of 98 °C for 10 s, 55 °C for 30 s, and 72 °C for 45 s; and a final extension at 72 °C for 10 min [58]. Amplicons were purified, quantified (Qubit 3.0), indexed, pooled equimolarly, and sequenced on an Illumina NovaSeq 6000 (paired-end 2 × 250 bp) following standard high-throughput amplicon sequencing workflows [59,60]. All pre-PCR and post-PCR procedures were physically segregated; batches were logged, and libraries were prepared and sequenced in a single run to limit cross-batch effects.

### 2.4. Read Processing and Taxonomy

Demultiplexed reads were processed in QIIME 2 (v2022.2) as implemented in [60]. The primers were removed with cutadapt, followed by quality filtering and trimming. DADA2 was subsequently used for denoising, paired-end merging, and chimera removal to infer amplicon sequence variants (ASVs) [59]. Taxonomic assignment employed Naive Bayes classifiers trained on SILVA 138 (16S) and the stable UNITE release (ITS) as described by Quast et al. [61]. Feature tables and representative sequences were retained for downstream analyses. For 16S analysis, sequences were aligned with MAFFT [62], and an approximately maximum-likelihood phylogeny was inferred with FastTree to obtain UniFrac metrics [63].

### 2.5. Diversity and Ordination

ASV tables were rarefied to the dataset-specific minimum depth (ITS: 29,848; 16S: 35,080 reads per sample). The α diversity indices included the observed ASVs and the Chao1, Shannon, and Simpson indices [59,64,65]. Rank–abundance plots were derived from the rarefied tables; group rank–abundance curves represent the median across biological replicates. For β diversity, counts were Hellinger-transformed (square root of relative abundance), and Bray–Curtis dissimilarities were computed on relative abundance and Hellinger-transformed data. PCoA was used for ordinations on the basis of Bray–Curtis and dbRDA results [66]. All statistical inference was performed at the biological replicate level. Pairwise distance heatmaps displayed Bray–Curtis, unweighted UniFrac, and weighted UniFrac matrices; diamonds indicate group means. These heatmaps are presented for descriptive comparison only and were clustered for visualization. Abundance heatmaps were generated from Hellinger-transformed matrices; rows were z scored for display. Additional visualizations included stacked bar plots at multiple taxonomic ranks, dendrogram–bar plots of the top 20 taxa per niche, Circos diagrams based on group means, and UpSet intersections at the phylum, family, and genus levels. For readability, some panels present group-level summaries aggregated from replicate data; all inferences were performed at the biological-replicate level.

### 2.6. Functional Prediction

Predicted bacterial functions were inferred with PICRUSt2 (v2.4.2), including 16S copy-number normalization and projection to KEGG Orthologs, Enzyme Commission numbers, KEGG/MetaCyc pathways, and COG categories [67]. Tax4Fun2 (v1.1) was additionally run using its default mapping to SILVA RefNR99 as implemented by the package [46]. Bacterial ecological functions were summarized with FAPROTAX (v1.2.4) [68]. Fungal ecological guilds were summarized with FUNGuild (Guilds_v1) [69]. The predicted-function outputs are presented as grouped comparisons and row-wise z score heatmaps.

### 2.7. Statistical Analysis

All analyses were performed in R (4.2) using vegan, phyloseq, microbiome, zCompositions, ggplot2, Metastats, UpSetR, and igraph [66,70,71,72]. α-Diversity was compared with the Kruskal–Wallis test with a BH-FDR control (*q* < 0.05) and pairwise Wilcoxon tests where appropriate. Different letters above boxplots indicate post hoc significance groupings (BH-FDR–adjusted pairwise Wilcoxon tests, *q* < 0.05); groups sharing a letter are not significantly different, whereas groups with no letters in common differ significantly. (Letter assignment is a statistical grouping label and does not indicate ranking by magnitude.) β-Diversity was evaluated by PERMANOVA and PERMDISP. With respect to differential abundance (phylum/family/genus), total-sum–scaled tables were used after low-information taxa were filtered; phylum/family contrasts were tested with Metastats (BH-FDR), and genus contrasts were performed with two-sided Wilcoxon (BH-FDR) tests. Effect sizes are reported as Δmedian values in percentage points and log2-fold-changes of group means, with 10,000-bootstrap 95% CIs. Genus co-occurrence networks were inferred from group-mean genus tables (prevalence ≥ 20%, global mean ≥ 0.01%); Spearman correlations with BH-FDR retained edges with |*ρ*| > 0.60 and *q* < 0.05 that recurred in ≥70% of 1000 bootstraps; visualization was performed with igraph (circle, Fruchterman–Reingold, sphere).

## 3. Results

### 3.1. Sample Groups and Sequencing Depth

ITS: CNW1, GJ1, K1, K2, K3, K4. 16S: CNW1, GJ1, TY1, TY2, TY3, T4. Six ecological niches were studied, with three biological replicates for each (*n* = 3). The definitions of these abbreviations are as follows: CNW1: parasitoid wasp *Eurytoma aethiops*; GJ1: parasitoid wasp *Aprostocetus* sp.; K1/TY1: healthy willow leaf surfaces (epiphytic microbiota); K2/TY2: willow gall surfaces (epiphytic microbiota); K3/TY3: gall interiors; K4/T4: host larval internal tissues (mouthparts + gut).

**Figure 4:**
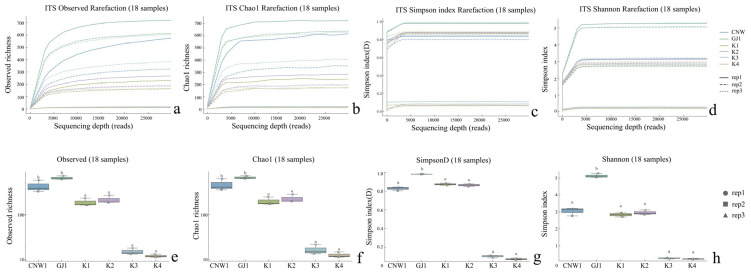
Alpha diversity across niches (ITS: (**a**–**h**)). Rarefaction curves for the observed, Chao1, Simpson, and Shannon indices; *p* < 0.05; Letters (*a*, *b*) indicate post hoc significance groups; groups sharing a letter are not significantly different.

**Figure 5:**
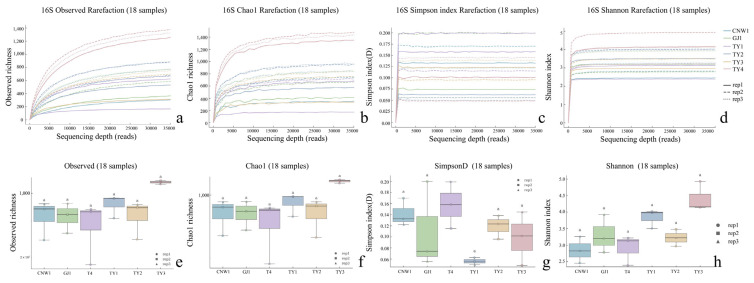
Alpha diversity across niches (16S: **a**–**h**). Rarefaction curves for the observed, Chao1, Simpson, and Shannon indices; *p* < 0.05; Letters (*a*) indicate post hoc significance groups; groups sharing a letter are not significantly different.

**Figure 6:**
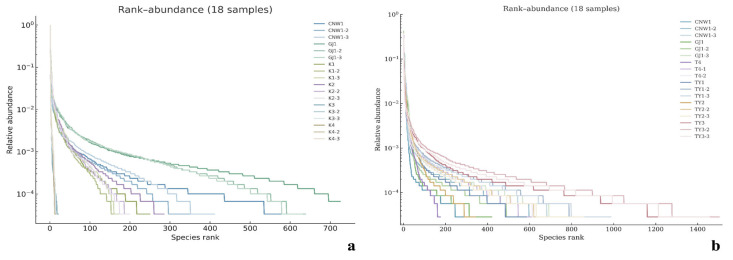
Rank–abundance (Whittaker) curves across niches. (**a**) ITS; (**b**) 16S. X-axis: species rank; Y-axis: relative abundance (log scale).

Across the 36 amplicon libraries included in this research (18 ITS1 and 18 V3–V4; six niches × three replicates), each dataset achieved adequate depth and replication (Figure 4a–h). For fungi (ITS1), sequencing produced 1,470,231 paired-end reads; after quality control, denoising and chimera removal, 1,352,160 high-quality reads were retained. The post-chimera cumulative depths per niche were 180,537 (CNW1), 89,544 (GJ1), 194,481 (K1), 298,746 (K2), 284,124 (K3) and 304,728 (K4), indicating stable coverage across plant/gall tissues and parasitoids. Taxonomic inference yielded 1673 ITS ASVs. Niche-level richness was 621 ± 33 for CNW1, 736 ± 54 for GJ1, 248 ± 15 for K1, 288 ± 23 for K2, 21 ± 2 for K3, and 15 ± 1 for K4, revealing high fungal richness on parasitoids, moderate richness on leaf/gall surfaces, and consistently low richness in gall interiors and host internal tissues. α-Diversity comparisons were performed at a standardized depth of 29,848 reads/sample (Figure 4a–h). For bacteria (16S V3–V4), quality-filtered, denoised and dechimerized libraries were standardized to 35,080 reads/sample for α diversity (a total of 631,440 reads across 18 libraries), yielding 3388 nonzero 16S ASVs. The unions of observed ASVs per niche (three replicates pooled) were TY3: 1983; TY1: 1488; TY2: 1294; CNW1: 1319; GJ1: 1280; T4: 1176; and niche-unique ASVs numbered 227 (TY3), 104 (TY1), 58 (TY2), 68 (GJ1), 64 (CNW1), and 34 (T4). The per-replicate richness values at the standardized depth (mean ± range) were TY3: 1444 ± 128, TY1: 848 ± 401, TY2: 658 ± 516, T4: 533 ± 554, GJ1: 654 ± 438, and CNW1: 660 ± 502 (Figure 5). Rank–abundance profiles corroborated these patterns: TY3 displayed the shallowest slope and longest tail (numerous rare taxa, lower evenness), TY1/TY2 presented intermediate values, and parasitoids (CNW1/GJ1) together with T4 presented steeper, short-tailed curves, and the ITS rank–abundance curves presented long tails for parasitoids and steep, short-tailed profiles for K3/K4 (Figure 6).

### 3.2. Results—α-Diversity

The sequencing depth was adequate for both the ITS and 16S datasets: the rarefaction curves reached clear plateaus for the Shannon and Simpson indices at low read counts and approached saturation for richness at greater depths (ITS ≈15–20k reads; 16S ≈20–30k reads), with consistent trajectories among biological replicates (Figure 4 and Figure 5). All downstream α diversity comparisons were therefore made at standardized depths (ITS: 29,848 reads; 16S: 35,080 reads per sample).

With respect to fungi (ITS), parasitoid niches presented the greatest within-sample diversity and richness, whereas enclosed larval niches were depleted. At standardized depth, the greatest Shannon and Simpson index values (higher evenness) and high richness values (observed and Chao1) were detected in GJ1, followed by those in CNW1. In contrast, K3 (gall interior) and K4 (host larval internal tissues) had markedly lower diversity and richness (Figure 4). Groupwise testing supported these differences; overall, the results of the Kruskal–Wallis tests were significant for all four indices (letters above boxplots indicate post hoc significance groupings; groups sharing a letter are not significantly different at BH-FDR *q* < 0.05, whereas groups with no letters in common differ significantly), and pairwise Wilcoxon contrasts (BH-FDRs) consistently separated parasitoids from K3/K4 (*q* < 0.05). Rank–abundance profiles corroborated these patterns, with GJ1 displaying the longest tails (many low-abundance taxa and high evenness) and K3/K4 showing steep, short-tailed curves indicative of dominance by few taxa (Figure 6a). For bacteria (16S), richness was greatest in gall interiors and lowest in parasitoids, whereas evenness showed the opposite trend between some niches. At standardized depth, richness (Observed/Chao1) ranked as TY3 > TY1 ≈ TY2 > T4 > GJ1 ≈ CNW1, whereas Simpson (1–D) indicated higher evenness in T4 and TY1 than in TY3 (Figure 5). Overall, the results of the Kruskal–Wallis tests for each index were significant, and post hoc Wilcoxon tests (BH-FDR) revealed differences that matched the letter groupings (q < 0.05). The 16S rank–abundance plot reinforced these findings: TY3 presented the shallowest slope and longest tail (numerous rare taxa, reduced evenness), TY1/TY2 presented intermediate slopes, and parasitoids (CNW1/GJ1) presented steep declines with short tails (low richness and strong dominance) (Figure 6b).

The results revealed contrasting niche filters for fungi and bacteria in the tritrophic system: parasitoids harbored rich and even fungal communities but species-poor bacterial communities, gall interiors harbored rich bacterial assemblages with lower evenness, and the internal larval niche was particularly fungus-depleted. These trends were robust across replicates and consistent between rarefaction, standardized-depth summaries, and rank–abundance diagnostics.

### 3.3. Results—β Diversity

**Figure 7:**
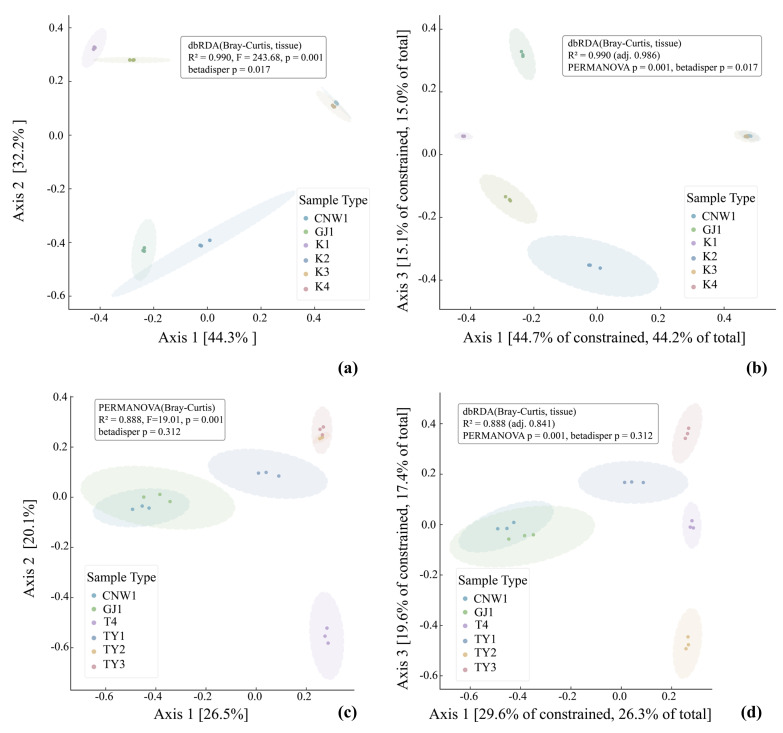
Beta diversity across niches (ITS: (**a**,**b**); 16S: (**c**,**d**)). (**a**,**c**) Bray–Curtis PCoA with group ellipses; (**b**,**d**) tissue-constrained dbRDA.

Bray–Curtis PCoA resolved a coherent niche structure in both marker datasets (Figure 7a,c). In the ITS ordination (Axis 1 = 44.3%, Axis 2 = 32.2%; Figure 7a), the two parasitoids—CNW1 (*E. aethiops*) and GJ1 (*Aprostocetus* sp.)—clustered tightly with partial overlap, indicating highly similar fungal assemblages. The two epiphytic plant niches (K1, healthy leaf surface; K2, gall surface) also grouped together with modest separation, whereas the gall interior (K3) and host larval internal tissues (K4) were distinctly isolated from all the other niches, which was consistent with strong compositional filtering in enclosed microhabitats. PERMANOVA supported a pronounced tissue effect (*R*^2^ = 0.990, *F* = 236.68, *p* = 0.001). Differences in multivariate dispersion were detected (betadisper *p* = 0.017), suggesting that unequal within-group variance modestly accentuated ITS separation. The tissue-constrained dbRDA (Figure 7b) recapitulated and sharpened the centroid differences (constrained *R*^2^ = 0.990; adj. *R*^2^ = 0.986), preserving the neighborhood structure of parasitoids (nearest neighbors), epiphytic surfaces (K1≈K2), and the two enclosed habitats (K3, K4) as endpoints. The 16S data exhibited the same hierarchy but with a clearer compositional basis (PCoA Axis 1 = 26.5%, Axis 2 = 22.9%; Figure 7c): parasitoids again formed a compact cluster; TY1 (leaf surface) and TY2 (gall surface) were adjacent with partial overlap; TY3 (gall interior) separated from surfaces; and T4 (host internal tissues) was most distinct. The PERMANOVA results remained significant (*R*^2^ = 0.888, *F* = 19.01, *p* = 0.001), whereas dispersion was homogeneous (betadisper *p* = 0.312), indicating that the 16S structure primarily reflected shifts in community centroids rather than variance artifacts. dbRDA constrained by tissue (Figure 7d) yielded congruent patterns with high explanatory power (constrained *R*^2^ = 0.889; adj. *R*^2^ = 0.841).

**Figure 8:**
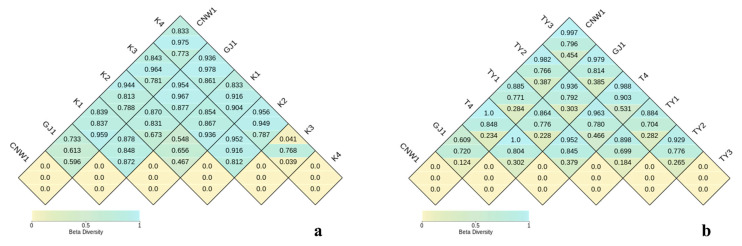
Pairwise β-diversity heatmaps (ITS: (**a**); 16S: (**b**)). Cells show between-group dissimilarity (0–1); darker colors indicate greater differences.

Pairwise distance heatmaps (Figure 8a,b) reinforced these conclusions. From top to bottom, each diamond indicates the Bray–Curtis, unweighted UniFrac and weighted UniFrac distances. In the ITS analysis (Figure 8a), CNW1–GJ1 had the smallest between-group distances, K1 and K2 had an intermediate distance, and both K3 and K4 were maximally distant from surface niches. With respect to 16S (Figure 8b), the same gradient held; moreover, weighted UniFrac values were generally lower than unweighted values, implying that phylogenetic turnover was driven by presence/absence more than by shifts in dominant lineages. Collectively, fungi and bacteria displayed a parallel β-diversity organization: parasitoid niches were most similar; plant surfaces were similar but nonidentical; and enclosed habitats (gall interior, host gut) harbored the most divergent communities—more pronounced for ITS (with some dispersion effects) and cleanly compositional for 16S.

### 3.4. Results—Community Composition

Across markers and taxonomic ranks, the community structure followed a consistent ecological hierarchy that mirrored α/β-diversity patterns: the two parasitoid niches were most alike; the leaf- and gall-surface microbiotas were similar but nonidentical; and enclosed habitats (gall interior and host larval tissues) harbored the most specialized assemblages.

**Figure 9:**
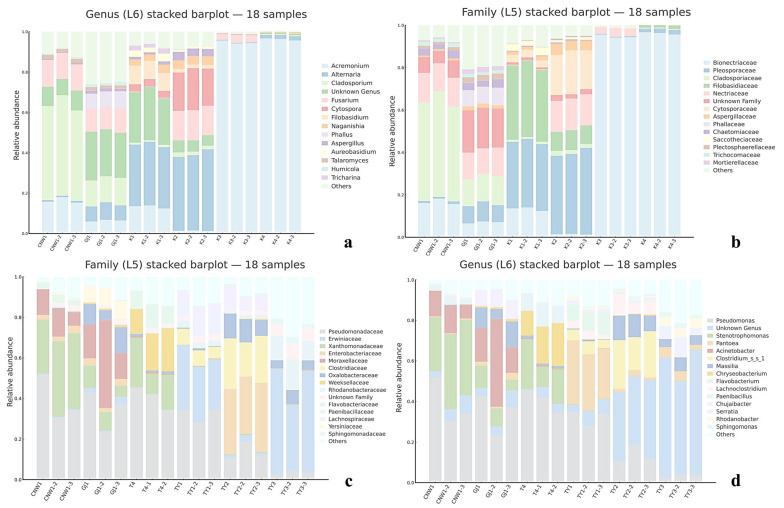
Community composition by niche (ITS: (**a**,**b**)); 16S: (**c**,**d**). Stacked bar plots at the family (**a**,**c**) and genus (**b**,**d**) levels; bars are samples grouped by niche.

**Figure 10:**
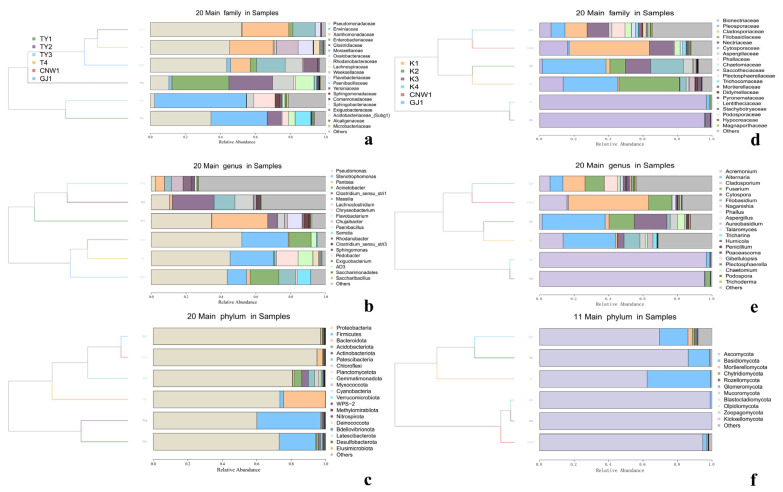
Dendrogram–bar plots by niche (ITS: (**a**–**c**); 16S: (**d**–**f**)). UPGMA trees (left) and stacked bars (right) show the relative abundance of the top taxa (family/genus), with branch lengths reflecting compositional similarity among niches.

**Figure 11:**
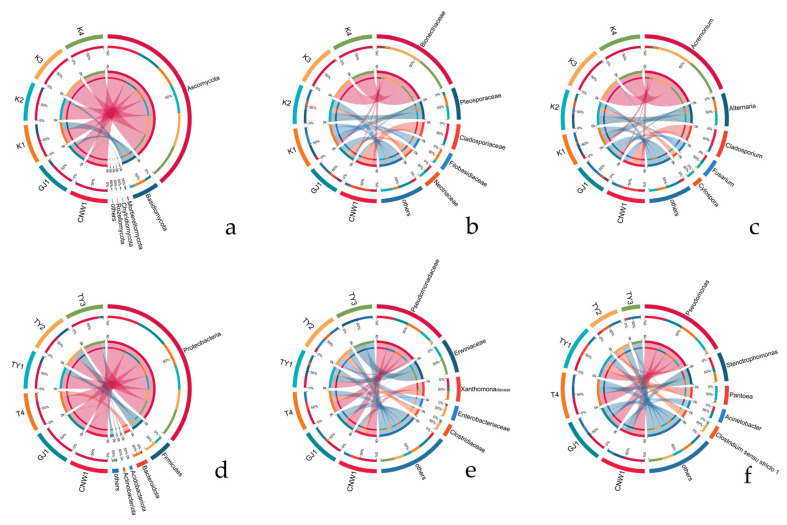
Circos chord diagrams of dominant genera across niches (ITS: (**a**–**c**); 16S: (**d**–**f**)). Ribbons indicate cross-niche sharing; outer rings show each niche’s relative abundance contribution.

**Figure 12:**
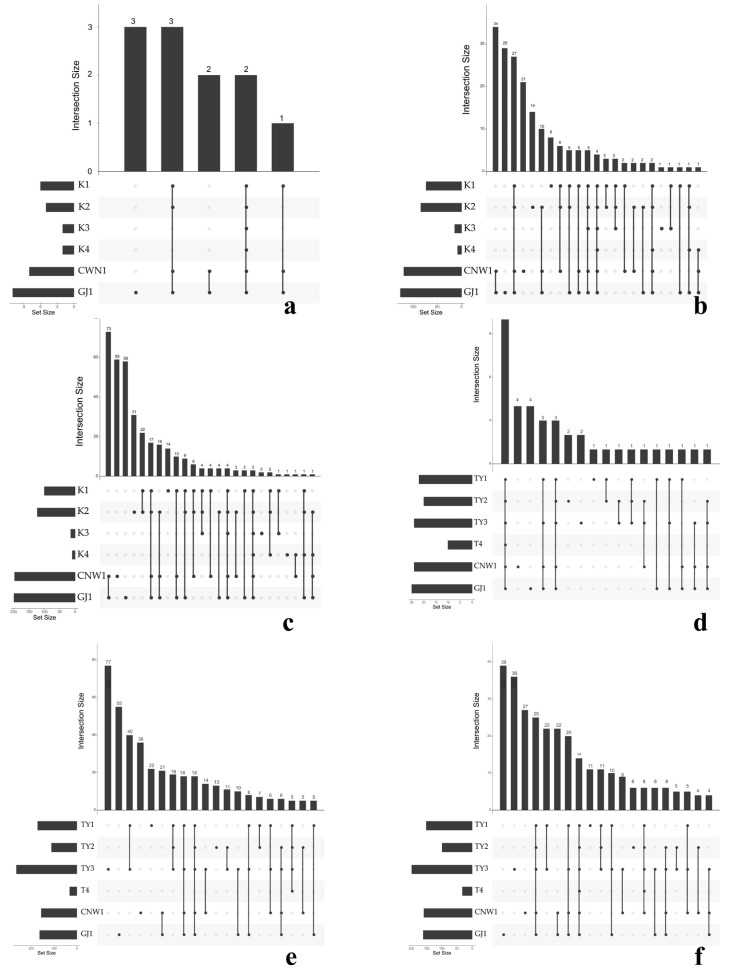
UpSet intersections across niches (ITS: (**a**–**c**); 16S: (**d**–**f**); ranks: phylum, family, genus). Top bars = intersection size; left bars = set size; the dot matrix indicates contributing niches.

At the phylum level, Ascomycota dominated all the niches, with a minor contribution of Basidiomycota (Figure 9a). Family- and genus-level profiles (Figure 9a,b and Figure 10a–c) resolved niche-specific enrichments superimposed on this shared backbone. Parasitoids (CNW1, GJ1) showed broadly similar compositions, with high representation of the pleosporalean and hypocrealean families (e.g., Pleosporaceae, Cladosporiaceae, Nectriaceae) and their genera (*Alternaria, Cladosporium, Fusarium*). The two plant-surface niches (K1 and K2) clustered together and were enriched for phyllosphere-associated families (Pleosporaceae/Cladosporiaceae), whereas the gall interior (K3) and host internal tissues (K4) were depauperate at the genus level and dominated by a restricted set of filamentous Ascomycetes, which was consistent with strong environmental filtering in enclosed microhabitats (Figure 12a–c and Figure S1a–c). Circos chord diagrams (Figure 11a–c) illustrate this division of labor: most high-abundance genera contributed across parasitoids and surfaces, whereas K3/K4 contributed disproportionately to a small subset of taxa. Pairwise Metastats tests (BH-FDR within contrast) supported these shifts at the phylum and family levels (*q* < 0.05), with the largest effect sizes observed for contrasts involving K3/K4 versus surfaces.

Proteobacteria were ubiquitous and often dominant, accompanied by Firmicutes, Actinobacteria, and Bacteroidetes in varying proportions (Figure 9c). The family/genus resolution (Figure 9c,d and Figure 10d–f) revealed clear niche signatures. Parasitoids again clustered together and were *Pseudomonas*-rich (Pseudomonadaceae). The two surfaces (TY1 leaf; TY2 gall) shared large fractions of Sphingomonadaceae and Comamonadaceae (e.g., *Sphingomonas, Massilia*), which was consistent with a phyllosphere imprint. The gall interior (TY3) was distinguished by Enterobacteriaceae/Xanthomonadaceae and genera such as *Pantoea* and *Serratia*, together with *Clostridium sensu stricto 1*, whereas host tissues (T4) were characterized by *Stenotrophomonas* and *Paenibacillus*. Heatmaps with hierarchical clustering (Figure S1d–f) and dendrogram–bar plots of the top 20 taxa (Figure 10d–f) recapitulated these groupings and emphasized within-cluster coherence. With respect to the ITS region, pairwise Metastats analysis revealed significant phylum-level and family-level differences (q < 0.05), especially for TY3 and T4 compared with the surface microbiotas.

UpSet summaries (Figure 12a–f) were obtained to quantify the balance between common cores and niche-specific sets. At higher ranks (phylum), the largest intersection spanned all six niches for both markers, reflecting the universal dominance of Ascomycota (ITS) and Proteobacteria (16S). At finer ranks (family/genus), the size of shared sets declined and that of unique sets increased, most notably for the gall interior and host tissues, confirming the strong specialization of enclosed habitats. Parasitoids retained a large shared component at all ranks, and TY1–TY2 shared the largest surface-associated set, which was consistent with their partial overlap in β diversity (Figure 7 and Figure 8).

Taken together, the stacked bars (Figure 9), Circos flows (Figure 11), clustered heatmaps (Figure S1), dendrogram–bar plots (Figure 10), and UpSet summaries (Figure 12) depicted a coherent compositional hierarchy. Parasitoid-associated microbiotas were compositionally similar; plant surfaces shared a phyllosphere-like consortium with moderate divergence between leaves and galls; and enclosed niches (the gall lumen and host digestive tissues) were the most compositionally distinct, driven by a narrowed set of dominant taxa. These results were fully concordant with those of the α-/β-diversity analyses and provided candidate taxonomic drivers for downstream differential and functional interpretation.

### 3.5. Results—Differentially Abundant Taxa

At the phylum level, the ITS analysis revealed 49 significant entries across 15 pairwise contrasts (2–7 in each). Ascomycota and Basidiomycota were significant in all comparisons and had the greatest effects (mean |*Δ*| = 17.51 and 16.29 percentage points, respectively) but presented opposing trends with respect to habitat openness: the abundance of Ascomycota was greater in K3/K4 than in CNW1/GJ1 and K1/K2, whereas the abundance of Basidiomycota was greater in surface niches; the parasitoid-associated symbiotic microbiota (CNW1 vs. GJ1) already contained seven significant phyla (Figure S2). The corresponding 16S profile retained 171 significant entries (5–16 per contrast) dominated by Proteobacteria and Firmicutes (18.47 and 17.90 percentage points, respectively). Contrasts involving TY3 showed peak replacements: the abundance of Proteobacteria was greater for parasitoids, whereas that of TY3 was greater for Planctomycetota, Cyanobacteria, Verrucomicrobiota, Myxococcota and Bdellovibrionota; in the comparison of TY1 versus TY3, the abundance of Firmicutes was 21.12 percentage points greater in TY1. The overall enrichment totals were as follows: TY3 = 62, TY1 = 35, GJ1 = 25, CNW1 = 22, TY2 = 18, and T4 = 9 (Figure S3). At the family tier, ITS yielded 287 significant entries (3–43 per contrast), with high recurrence for Cladosporiaceae, Pleosporaceae, Bionectriaceae, Didymellaceae, Aspergillaceae, Nectriaceae, Chaetomiaceae, Filobasidiaceae and Hypocreaceae. Within the parasitoid-associated symbiotic microbiota, CNW1 favored Cladosporiaceae, whereas GJ1 favored Pleosporaceae/Didymellaceae; open surfaces (K1/K2) presented values exceeding those of enclosed or host-internal sites (K3/K4) for Cladosporiaceae/Aspergillaceae/Aureobasidiaceae (Figure S4). The corresponding 16S data showed directional replacement along the host–habitat gradient, with 580 significant entries (13–93 per contrast); Pseudomonadaceae recurred in all the contrasts (mean |*Δ*| ≈ 25.12 percentage points), and Erwiniaceae had the greatest mean effect (~29.64; 10/15), with Xanthomonadaceae, Enterobacteriaceae and Oxalobacteraceae/Moraxellaceae/Lachnospiraceae/Rhodanobacteraceae contributing. The abundance of Pseudomonadaceae was greater on TY1 and on parasitoids, whereas Erwiniaceae/Sphingomonadaceae/Comamonadaceae was consistently greater on TY3 (TY3 ≈ 269 > TY1 ≈ 111 > TY2 ≈ 71 > GJ1 ≈ 57 > CNW1 ≈ 37 ≈ T4 ≈ 35) (Figure S5). At the genus level, ITS analysis recovered 327 significant entries (3–81 per contrast), where the dominant, high-effect genera were *Cladosporium*, *Alternaria*, *Acremonium* and *Fusarium* (mean |*Δ*| ≈ 51.9, 25.8, 22.4 and 10.3 percentage points, respectively). A consistent split within the parasitoid-associated symbiotic microbiota was evident (CNW1 enriched for *Cladosporium*/*Acremonium*; GJ1 enriched for *Alternaria* ± *Phallus*), and open surface (K1/K2) values exceeded those of enclosed or host-internal sites (K3/K4) for surface-associated genera (Figure S6). The corresponding 16S layer identified 514 significant genus-by-contrast entries (9–85 per contrast); high-recurrence lineages included *Massilia*, *Pseudomonas*, *Stenotrophomonas* (14/15), *Bryobacter* (12/15), KD4-96 (placeholder; not italicized) and *Sphingomonas* (10/15). The greatest effects were observed for *Pseudomonas* (~27.3 percentage points), *Pantoea* (~17.8), *Stenotrophomonas* (~16.4) and *Acinetobacter* (~10.6). The abundance of *Pseudomonas* was greater on TY1 and on parasitoids, whereas that of *Erwinia*/*Sphingomonas*/*Comamonas* was greater on TY3; the enrichment totals were as follows TY3 = 220 > GJ1 = 102 > TY1 = 84 > TY2 = 45 > T4 = 34 > CNW1 = 29 (Figure S7).

### 3.6. Results—Co-Occurrence Networks

Genus-level networks were inferred from filtered TSS-normalized group profiles (prevalence ≥20%, mean relative abundance ≥0.01%). Spearman correlations were BH–FDR adjusted; edges with |*ρ*| > 0.60 and *q* < 0.05 were retained only if reproduced in ≥70% of 1000 bootstrap replicates. Species correlation networks were plotted in R using the *igraph* package and displayed in three layouts (Figure 12): circle (global symmetry/grouping), Fruchterman–Reingold (module structure in denser graphs), and sphere (fewer edge crossings and clearer separation for larger node sets).

**Figure 13:**
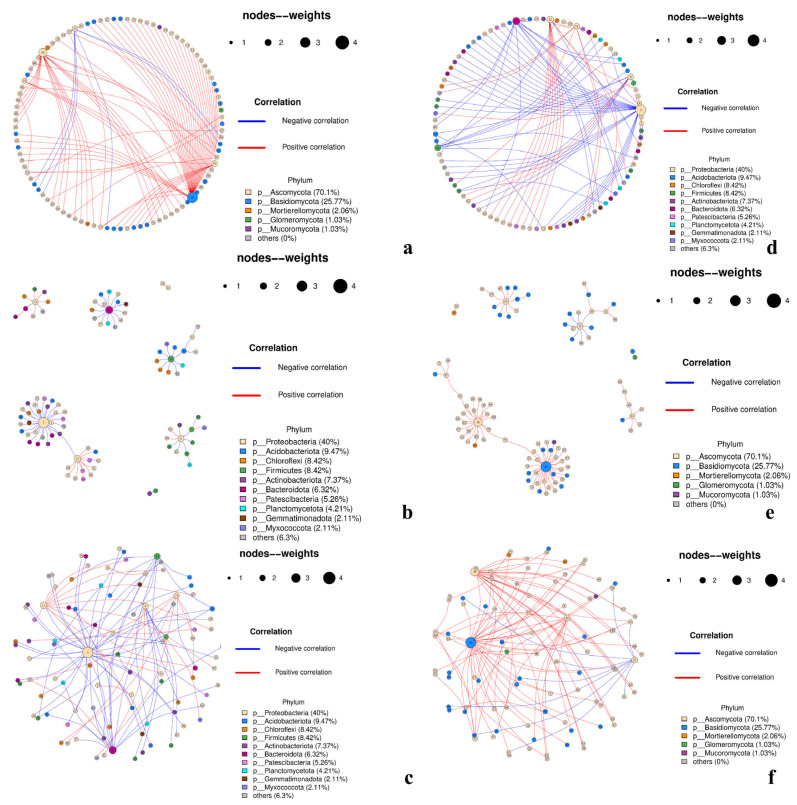
Co-occurrence networks (ITS: (**a**–**c**); 16S: (**d**–**f**)). Nodes are colored by phylum and scaled by degree/abundance; edges show correlations (red, positive; blue, negative). Three layouts (circular, modular, and force-directed) highlight hubs and modules.

The ITS network (Figure 13a–c) comprised 97 nodes and 97 edges (mean degree 2.0; density 0.0208; modularity 0.767), with a high percentage of positive associations (≈0.90). Degree and betweenness centralization were moderate (≈0.31 and ≈0.27, respectively), the clustering coefficient was near zero, and the average shortest path in the largest component was ≈3.12. The graph is resolved into several compact modules linked by a small number of bridge hubs. High-centrality genera included the basidiomycete genus *Phallus* (*k* = 32) and the ascomycetes *Cladosporium* (*k* = 22), *Acremonium* (*k* = 10), and *Alternaria* (*k* = 10), with *Cytospora* (*k* = 6) and *Penicillium*, *Talaromyces*, *Coniochaeta*, and *Tricharina* (each *k*≈3) acting as secondary connectors. The predominance of positive edges indicated co-occurrence or shared environmental filtering. Module composition aligned with the ecological structure described above: phyllosphere-associated ascomycetes cluster with parasitoid- and surface-enriched taxa, whereas enclosed habitats (gall interior, host tissues) contributed to smaller, more selective subgraphs connected via interface hubs. Overall, the ITS layer suggested a facilitative, guild-structured assembly maintained by a few cross-module bridges.

In contrast, the 16S network (Figure 13d–f) contained 95 nodes and 95 edges (mean degree 2.0; density 0.021) and exhibited similarly high modularity (0.814) and a much lower positive edge ratio (~0.32) than the ITS result indicating that negative associations predominated. Centralization was modest (degree ≈0.29; betweenness ≈0.15), and the average shortest path was ≈2.33. A small set of Proteobacteria-rich hubs organized the graph across niches, most prominently for *Pseudomonas* (*k* = 29), with *Chryseobacterium* (Bacteroidota; *k* = 15), *Chujaibacter* (*k* = 12), *Exiguobacterium* (Firmicutes; *k* = 10), *Acinetobacter* (*k* = 9), and *Pantoea* (*k* = 8) as additional hubs, while *RB41* (Acidobacteriota; *k* = 4) and *Clostridium sensu stricto* 1 (Firmicutes; *k* = 4) served as subhubs. Blue (negative) edges frequently spanned modules corresponding to surface/parasitoid versus gall-interior/host-internal niches, which was consistent with β diversity and differential abundance contrasts (TY1/TY2/CNW1/GJ1 vs. TY3/T4). Within modules, positive edges were common among phyllosphere/parasitoid taxa (often Proteobacteria-dominated), whereas negative ties delineated habitat-specific replacements, indicating pronounced niche partitioning and putative competitive exclusion in bacteria.

Despite having similar sparsity and strong modularity, the two networks revealed distinct assembly mechanics. The ITS graph was dominated by positive, within-guild links and a handful of interface hubs (most notably for *Cladosporium*, *Alternaria*, and *Acremonium*) that bridged surface/parasitoid-enriched fungi with enclosed-habitat subsets. In contrast, the 16S graph was structured by hub-mediated, often negative ties, with *Pseudomonas*, *Stenotrophomonas*, *Acinetobacter*, and *Pantoea* organizing habitat-defined modules and marking high-effect replacements between surface/parasitoid and gall-interior/host-internal niches. These network-level results reinforced the α/β-diversity and differential-taxa findings and suggested a compact set of candidate keystone genera for follow-up validation.

### 3.7. Results—Predicted Functions

Putative functional profiles were inferred from marker–gene data (FUNGuild (ITS) for fungi and PICRUSt2, Tax4Fun2, and FAPROTAX (16S) for bacteria) recognizing that these are association-based predictions rather than metagenome-resolved functions and are most appropriately interpreted compared across niches.

**Figure 14:**
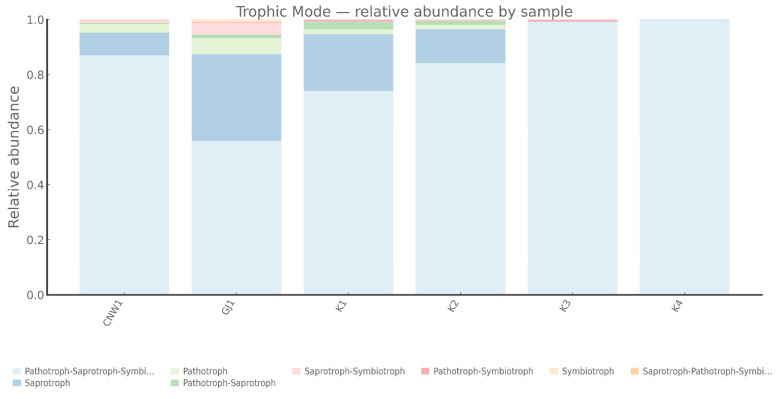
Fungal trophic modes across niches (ITS; FUNGuild).

**Figure 15:**
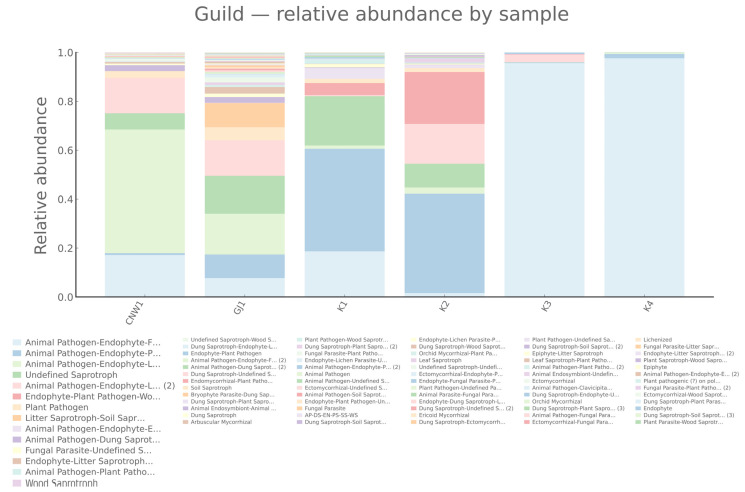
Fungal guild composition across niches (ITS; FUNGuild).

FUNGuild assigned fungal ASVs to pathotrophs, symbiotrophs, and saprotroph guilds with clear habitat signatures (Figure 14 and Figure 15). Pathotrophs—especially the *Endophyte–Plant Pathogen* and *Plant Pathogen* guilds—were enriched on the gall surface and within galls, indicating stronger coupling between gall-associated fungi and plant health in enclosed or semi-enclosed microhabitats. Symbiotrophs, dominated by *Arbuscular Mycorrhizal* annotations, prevailed in host internal tissues, which was consistent with the idea that symbiosis-leaning interactions are likely shaped by plant-derived substrates. Saprotrophs were most abundant on parasitoid wasps (*Eurytoma aethiops* and *Aprostocetus* sp.), reflecting organic matter turnover on exposed hosts. Notably, host internal tissues were dominated by Undefined Saprotrophs, while the gall lumen exhibited a mixed pathotroph–saprotroph structure, suggesting more complex functional roles inside the gall.

**Figure 16:**
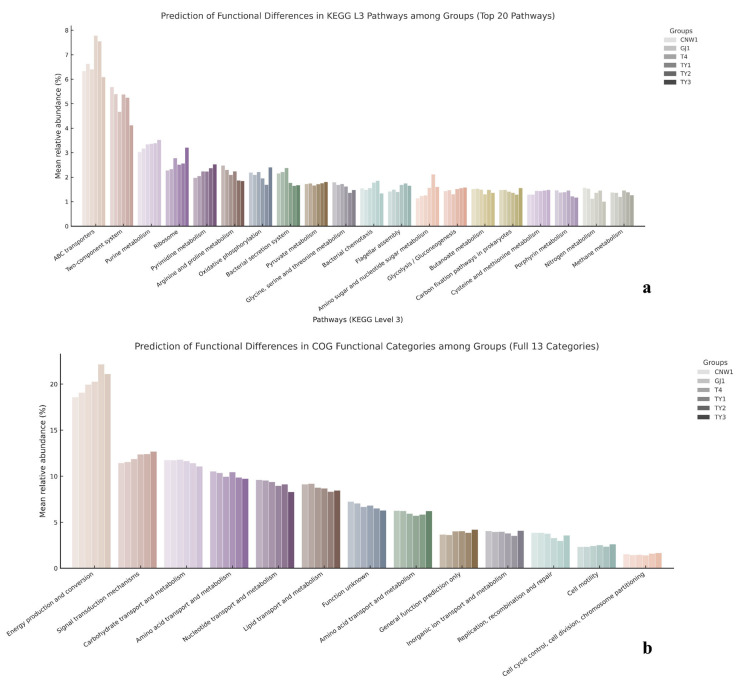
Predicted bacterial functions from 16S (PICRUSt2). (**a**) KEGG level-3 pathways (top 20) summarized by group; (**b**) COG functional categories (13) summarized by group.

PICRUSt2 projections (copy-number–normalized 16S ASVs mapped to KEGG/COG) revealed a pronounced gradient from exposed niches—leaf and gall surfaces and parasitoids—through host tissues to the gall interior, as shown by grouped comparisons (Figure 16a,b) and heatmaps (row-wise z scores) (Figure S8a,b). KEGG L3 modules linked to transport, sensing, secretion, and motility (ABC transporters, two-component systems, bacterial secretion, and chemotaxis/flagellar assembly) as well as core growth/energy pathways (ribosome, oxidative phosphorylation, and pyruvate metabolism) were consistently elevated in exposed niches and attenuated in the gall interior. Clustering placed parasitoids with the gall surface at the high end of this axis, the leaf surface and host tissues at intermediate levels, and the gall interior at the lowest end, mirroring the β-diversity structure (Figure 16a). The results for COG categories converged with these trends: amino acid/carbohydrate transport and metabolism, energy production and conversion, and transcription were lower in the gall interior than in the parasitoid and surface regions, with the gall surface clustering closest to the parasitoid and the leaf surface adjacent to the surface-exposed group (Figure 16b).

**Figure 17:**
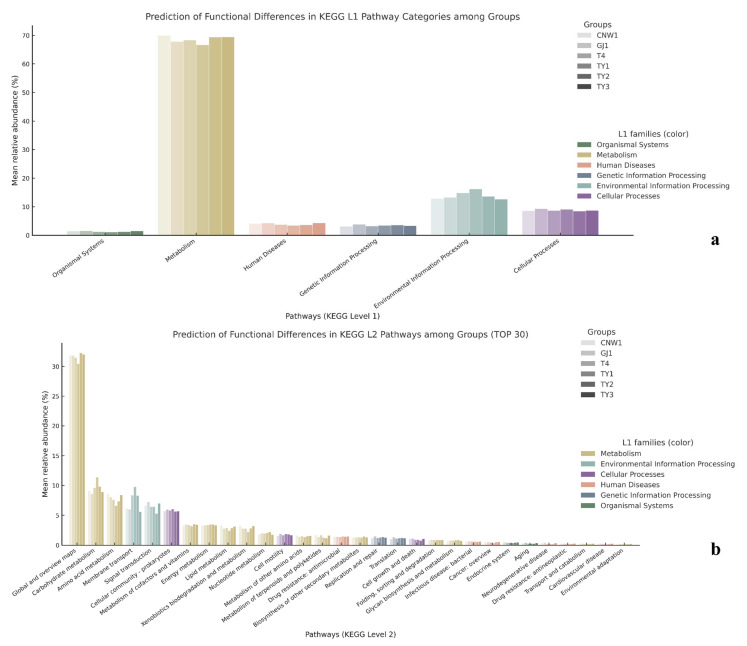
Predicted KEGG functions from 16S (PICRUSt2). (**a**) Level-1 pathway categories by group; (**b**) Level-2 pathways (top 30).

Tax4Fun2 (SILVA-based KEGG projection) corroborated these patterns at KEGG L1–L2, as shown by grouped comparisons (Figure 17a,b) and row-wise z score heatmaps (Figure S9). While metabolism dominated across niches, environmental information processing and cellular processes differentiated habitats: the leaf surface showed a relatively large information-processing share, parasitoids and the gall surface were skewed toward metabolism, and the gall interior exhibited decreased transport/motility alongside increases in categories that aggregate stress/virulence-related functions. At L2, membrane transport, signal transduction, cell motility, and the cellular community (prokaryotes) separated the exposed niches from the gall interior; translation and replication/repair peaked on one parasitoid, energy metabolism was elevated on the plant surface, carbohydrate metabolism was most pronounced on the leaf surface, amino acid metabolism was relatively high on one parasitoid and in the gall interior, and nucleotide metabolism was highest at the gall surface. Together, these results indicate distinct resource acquisition and growth strategies across habitats.

**Figure 18:**
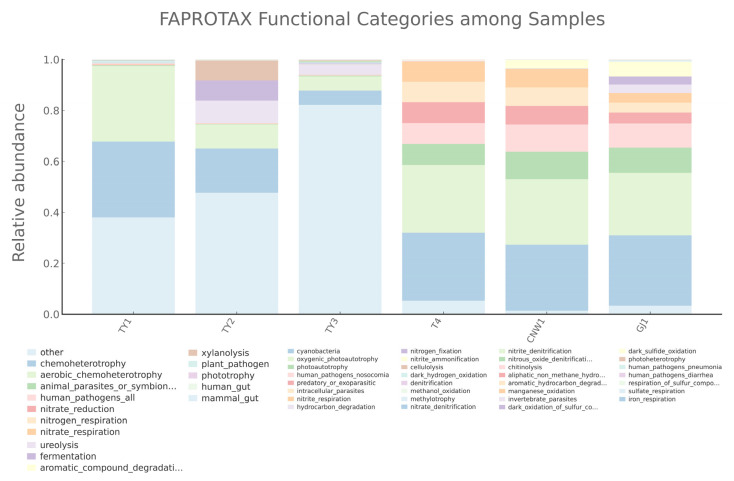
Predicted prokaryotic functions across 16S niches (FAPROTAX). Stacked bars show the relative abundance of FAPROTAX functional categories.

Literature-curated taxon-to-function mapping (FAPROTAX) further resolved ecological roles (Figure 18). Aerobic chemoheterotrophy dominated the parasitoid and plant surfaces, whereas the gall interior presented a relatively large “unmapped/other” fraction and attenuated classic heterotrophy labels. Nitrogen-respiration capacities distinguished host internal tissues and parasitoids from plant surfaces (nitrate/nitrite respiration was highest in host tissues and evident in parasitoids), whereas denitrification annotations were detectable in plant-associated niches but minimal in parasitoids and host tissues. Ureolysis was strongest at the gall surface and was present in the gall interior. The use of plant-surface polysaccharides was niche specific (xylanolysis at the gall surface; minor cellulolysis on the leaf surface), with only scattered cellulose/xylan tags in the gall interior. Parasitoids exhibited broader oxidative/degradative potential (aromatic/aliphatic hydrocarbon degradation; dark sulfide/sulfur oxidation), and manganese oxidation occurred in the gall interior. Phototrophy-related labels clustered on plant-associated niches and were minimal on parasitoids and host tissues, and plant–pathogen terms appeared on the leaf surface.

The predicted-function landscape recapitulated the compositional and β-diversity hierarchy: in exposed niches, transport, sensing, motility, and heterotrophy were highlighted; the gall interior showed attenuated translocation/growth modules with selective nitrogen/sulfur features; and host tissues presented information-processing and nitrogen-respiration signatures. These results give rise to testable hypotheses for targeted metagenomics/metatranscriptomics and enzyme-activity assays while acknowledging the inferential nature of function mapping.

## 4. Discussion

Different tissues and niches constitute plant–insect-associated microbiomes with regular functional biases [73]. Ecological niches differ in α/β diversity and indicator taxa, and functional categories are reorganized among tissues [74]. Microhabitat gradients of oxygen, UV, water potential, exudates, and immune tone screen for transport, sensing, secretion, and redox modules on exposed surfaces but increase fermentation, N resp, and polysaccharide utilization within covered niches [75]. In the pest–plant system, herbivores and their endosymbionts regulate volatiles and defense mechanisms, impacting colonization and feeding; phyllosphere core genera—*Sphingomonas*, *Pseudomonas*, and *Massilia*—are aligned through chemotaxis, two-component signaling, and adhesion, and endophytes prime defense and reorganize C/N pools [76,77]. In the pest–enemy system, host gut and cuticular microbial communities follow the parasitoid developmental stage, survival, and searching efficiency; semiochemicals and microbial metabolites determine host finding; and parasitic endosymbionts can alter host immunity upon oviposition [53,78]. Fungal associates such as *Cladosporium*, *Alternaria*, and *Acremonium* connect surface communities under disturbance, and vertical/horizontal transmission is associated with sustained turnover [79]. According to the most recent developments reported in these dyads, few investigations have examined pests, plants, natural enemies, and endosymbionts within a single analytical frame that integrates site, community, function, and behavior on one ecological axis [80,81].

Across the willow–gall–parasitoid complex, we identify a coherent open-to-closed habitat gradient: leaf and gall surfaces cluster with parasitoid-associated microbiomes, whereas gall lumina and host internal tissues anchor the opposite end of the axis. This organization recapitulates α- and β-diversity and predicted functional profiles, indicating that cross-trophic concordance is governed primarily by habitat context rather than host identity [30,82].

Environmental filtering and biotic interactions offer a succinct solution. Exposed niches with pulsed, oxygenated substrates select for *Pseudomonas*, *Sphingomonas*, and *Massilia*, matching high transport/two-component/secretion–motility scores; closed niches with diffusion and immune filters select for *Pantoea* and *Serratia*, with strong replacement signals. Both the architecture of the communities and their functions match these predictions: bacterial communities consist largely of negative edges indicative of habitat-specialized substitutions, with fungal communities dominated by positive edges between guilds connected by *Cladosporium*, *Alternaria*, and *Acremonium* species in fungal communities. The bacterial community structure around the *Pseudomonas*, *Acinetobacter*, and *Pantoea* hubs sharply differs between surface-exposed gall regions and more interior regions of the gall, reflecting differences in oxygenation, UV exposure, and substrate type, compared with the low-oxygen, immune-privileged interior [30,83,84].

The allocation of roles follows ecological reality. Surface fungal networks emphasize positive associations and module-level synchronization, where the leaf/gall surfaces and parasite subsets are connected by *Cladosporium*, *Alternaria*, and *Acremonium*. Bacterial modules are hub constructed by *Pseudomonas*, *Acinetobacter*, and *Pantoea*. They are compatible with the pulse of soluble substances under high-oxygen/UV, hydrophobic surface, boundary conditions, versus the viscous, low-oxygen, immune-privileged interior of galls.

We defined clear boundaries for our conclusions. Our sampling involved only one outbreak and was not conducted across different seasons or years. We examined only the larvae of *E. viminalis* and their beneficial microbes and not the microbes within infected larvae or their evolution throughout various stages. We sampled only one of the local *Salix* species, although *E. viminalis* occurs throughout much of the territory, and parameters such as height, sun, humidity, and the environment may influence interactions and outcomes. Our 16S/ITS and functional projection methods also suffered from certain limitations, such as copy number, primer, composition, and label problems. Network correlation analyses did not resolve specific interactions; within these enclosed microhabitats, relative patterns were prioritized, with contamination minimized by surface sterilization and aseptic handling. Future work should convert associations to mechanisms within the *Salix*-gall frame: season × interannual × altitude/irradiance/humidity/stand stratification; paired nonparasitized versus parasitized larvae with finer staging; shotgun/metatranscriptomic plus absolute-quantification validation of transport/two-component/secretion/nitrogen-cycling modules; an integrated GC–MS–behavior–core-taxa loop; and models that explicitly handle spatial autocorrelation and environmental covariates to partition environmental filtering from dispersal limitation.

## Data Availability

Raw high-throughput sequencing reads can be obtained from the NCBI Sequence Read Archive (SRA) under BioProject accession PRJNA1354980, or from the corresponding author upon reasonable request.

## Supplementary Materials

The following supporting information can be downloaded at: https://www.mdpi.com/article/10.3390/insects17010043/s1, Figures S1–S9: Figure S1: Taxonomic heatmaps across niches (ITS: a–c; 16S: d–f). (a,d) Phylum; (b,e) Family; (c,f) Genus; Colors show row-scaled relative abundance with hierarchical clustering of taxa. Figure S2: Pairwise differential-abundance heatmaps for fungal phyla (ITS). Figure S3: Pairwise differential-abundance heatmaps for fungal phyla (16S). Figure S4: Pairwise differential-abundance heatmaps for family phyla (ITS). Figure S5: Pairwise differential-abundance heatmaps for family phyla (16S). Figure S6: Pairwise differential-abundance heatmaps for genera phyla (ITS). Figure S7: Pairwise differential-abundance heatmaps for genera phyla (16S). Figure S8: Functional prediction heatmaps (16S). (a) KEGG Level-3 top-30 pathways; (b) COG 13 functional categories. Figure S9: KEGG functional profiles (16S, PICRUSt2). (a) Heatmap of KEGG Level-1 categories; (b) heatmap of representative Level-2 pathways.

## References

[1] Cruaud A., Rasplus J.Y., Zhang J., Burks R., Delvare G., Fusu L., Gumovsky A., Huber J.T., Janšta P., Mitroiu M.D. (2024). The Chalcidoidea bush of life: Evolutionary history of a massive radiation of minute wasps. Cladistics.

[2] Haas M., Baur H., Schweizer T., Monje J.C., Moser M., Bigalk S., Krogmann L. (2021). Tiny wasps, huge diversity—A review of German Pteromalidae with new generic and species records. Biodivers. Data J..

[3] Koutsoukos E., Demetriou J., Georgiadis C., Mitroiu M., Compton S.G., Martinou A. (2024). Highlighting overlooked biodiversity through online platforms: The “Chalcid Wasps of Cyprus” website. Biodivers. Data J..

[4] Khudhair A.H., Kareem A.A., Watson G.W., Kresslein R.L., Beasley J., Topakcı N., Polaszek A. (2025). Parasitoids of the invasive cotton mealybug, *Phenacoccus solenopsis* Tinsley (Hemiptera, Pseudococcidae), in Iraq. BioControl.

[5] Jara-Chiquito J.L., Oliva F., Lobato-Vila I., Pujade-Villar J. (2024). Temporal changes in the composition of parasitoid assemblages associated with the invasive chestnut gall wasp. Ecol. Entomol..

[6] Jennings M.T., Askew R.R. (2020). Recruitment of native parasitoids by an introduced gall wasp *Dryocosmus kuriphilus* Yasumatsu, 1951 (Hymenoptera: Cynipidae) in Britain and France. Entomol. Mon. Mag..

[7] Hou E. (2021). Parasitic Hymenopteran food and habitat preferences in urban agriculture. Agric. Food Sci..

[8] Kos K., Lacković N., Melika G., Matošević D. (2021). Diversity and surge in abundance of native parasitoid communities prior to the onset of *Torymus sinensis* on the Asian chestnut gall wasp (*Dryocosmus kuriphilus*) in Slovenia, Croatia and Hungary. J. For. Res..

[9] Khamis F.M., Ajene I.J. (2025). Anthropogenic influences on parasitoid wasps’ biocontrol of invasive insect pest species in Africa. Curr. Opin. Insect Sci..

[10] Zhang X., Jiang Z., Jiao X., Yu Y., Wang Z., Hou Y., Duan G., Du W., Ruan C., Zhang J. (2023). Genome assembly and comparative analysis of the egg parasitoid wasp *Trichogramma dendrolimi* shed light on the composition and evolution of olfactory receptors and venoms. Insects.

[11] Del Pino M., Cabello T., Hernández-Suárez E. (2022). Biological control options for the golden twin-spot moth, *Chrysodeixis chalcites* (Esper) (Lepidoptera: Noctuidae) in Banana crops of the canary Islands. Insects.

[12] Kareem A.A., Lotfalizadeh H., Alsendi A., Aljaafari R.K., Al-Zurfi S.M. (2022). First record of two parasitoid wasps of the family Chalcididae (Hymenoptera) in Iraq. Bull. Iraq Nat. Hist. Mus..

[13] Saleh H.M.M., Dey D. (2024). New records of family Chalcididae (Hymenoptera: Chalcidoidea) on vegetables ecosystem from New Delhi, India. Orient. Insects.

[14] Smith E.L. (1970). Biosystematics and morphology of symphyta. Ii. Biology of gall-making nematine sawflies1 in the California region. Ann. Entomol. Soc. Am..

[15] Michell C., Wutke S., Aranda M., Nyman T. (2021). Genomes of the willow-galling sawflies *Euura lappo* and *Eupontania aestiva* (Hymenoptera: Tenthredinidae): A resource for research on ecological speciation, adaptation, and gall induction. G3 Genes|Genomes|Genet.

[16] Price P.W., Hunter M.D. (2015). Population dynamics of an insect herbivore over 32 years are driven by precipitation and host-plant effects: Testing model predictions. Environ. Entomol..

[17] Sacchi C.F., Price P.W., Craig T.P., Itami J.K. (1988). Impact of shoot galler attack on sexual reproduction in the arroyo willow. Ecology.

[18] Urban J. (1995). Mortalität der blattwespen-arten *Euura laeta* und *E. mucronata* infolge physiologischer ursachen und durch abwehr-überwallungswachstum der wirtspflanzen-gewebe (Symphyta: Tenthredinidae). Entomol. Gen..

[19] Roininen H., Danell K. (1997). Mortality factors and resource use of the bud-galling sawfly, *Euura mucronata* (Hartig), on willows (*Salix* spp.) in arctic Eurasia. Polar Biol..

[20] Kopelke J.-P. (2003). Natural enemies of gall-forming sawflies on willows (*Salix* spp.) (Hymenoptera: Tenthredinidae: *Euura*, *Phyllocolpa*, *Pontania*). Entomol. Gen..

[21] Craig T.P., Itami J.K., Price P.W. (1990). The window of vulnerability of a shoot-galling sawfly to attack by a parasitoid. Ecology.

[22] Roininen H., Price P.W., Tahvanainen J. (1996). Bottom-up and top-down influences in the trophic system of a willow, a galling sawfly, parasitoids and inquilines. Oikos.

[23] Dicke M., Cusumano A., Poelman E.H. (2020). Microbial symbionts of parasitoids. Annu. Rev. Entomol..

[24] Cusumano A., Zhu F., Volkoff A.N., Verbaarschot P., Bloem J., Vogel H., Dicke M., Poelman E.H. (2018). Parasitic wasp-associated symbiont affects plant-mediated species interactions between herbivores. Ecol. Lett..

[25] Wang J., Mason C.J., Ju X., Xue R., Tong L., Peiffer M., Song Y., Zeng R., Felton G.W. (2021). Parasitoid causes cascading effects on plant-induced defenses mediated through the gut bacteria of host caterpillars. Front. Microbiol..

[26] Pekas A., Tena A., Peri E., Colazza S., Cusumano A. (2023). Competitive interactions in insect parasitoids: Effects of microbial symbionts across tritrophic levels. Curr. Opin. Insect Sci..

[27] Triyogo A., Yasuda H. (2019). The effects of a parasitoid wasp of a gall-making insect on host plant characteristics and the abundance of sharing host-plant herbivore. Biodiversitas J. Biol. Divers..

[28] Fang Z., Tang C.T., Sinclair F., Csóka G., Hearn J., McCormack K., Melika G., Mikolajczak K.M., Nicholls J.A., Nieves-Aldrey J.L. (2024). Network structure and taxonomic composition of tritrophic communities of Fagaceae, cynipid gallwasps and parasitoids in Sichuan, China. Insect Conserv. Divers..

[29] De Araújo W.S., Maia V.C. (2021). Topological structure of a tritrophic network composed of host plants, gall-inducing insects and parasitoids in a restinga area in Brazil. Entomol. Sci..

[30] Michell C.T., Nyman T. (2021). Microbiomes of willow-galling sawflies: Effects of host plant, gall type, and phylogeny on community structure and function. Genome.

[31] Hansen A.K., Argondona J.A., Miao S., Percy D.M., Degnan P.H. (2024). Rapid loss of nutritional symbionts in an endemic Hawaiian herbivore radiation is associated with plant galling habit. Mol. Biol. Evol..

[32] Men Y., Yang Z., Luo J., Chen P., Moreira F.F.F., Liu Z., Yin J., Xie B., Wang Y., Xie Q. (2022). Symbiotic microorganisms and their different association types in aquatic and semiaquatic bugs. Microbiol. Spectr..

[33] Jones J.A., Newton I.G., Moczek A.P. (2025). Microbiome composition and turnover in the face of complex lifecycles and bottlenecks: Insights through the study of dung beetles. Appl. Environ. Microbiol..

[34] Baine Q., Hughes D.W.W., Casares E.E., Martinson E.O., Martinson V.G. (2024). External insect gall morphology influences the functional guilds of natural enemy communities. Proc. R. Soc. B Biol. Sci..

[35] Michell C.T., Wagner N., Mutanen M., Lee K.M., Nyman T. (2023). Genomic evidence for contrasting patterns of host-associated genetic differentiation across shared host-plant species in leaf- and bud-galling sawflies. Mol. Ecol..

[36] De Carvalho-Sposito S.H., Urso-Guimarães M.V., da Silva F.R. (2022). Temporal resource partitioning and stochastic colonization explain the co-occurrence of gall-inducing insects in the super-host plant *Copaifera langsdorffii* Desf. (Fabaceae). Austral Ecol..

[37] Dong Y., Li Y., Ge M., Takatsu T., Wang Z., Zhang X., Ding D., Xu Q. (2023). Distinct gut microbial communities and functional predictions in divergent ophiuroid species: Host differentiation, ecological niches, and adaptation to cold-water habitats. Microbiol. Spectr..

[38] Liu Y., Shen Z., Yu J., Li Z., Liu X., Xu H. (2020). Comparison of gut bacterial communities and their associations with host diets in four fruit borers. Pest Manag. Sci..

[39] Ingala M.R., Simmons N.B., Dunbar M., Wultsch C., Krampis K., Perkins S.L. (2021). You are more than what you eat: Potentially adaptive enrichment of microbiome functions across bat dietary niches. Anim. Microbiome.

[40] Xiong C., Singh B.K., He J.-Z., Han Y.-L., Li P.-P., Wan L.-H., Meng G.-Z., Liu S.-Y., Wang J.-T., Wu C.-F. (2021). Plant developmental stage drives the differentiation in ecological role of the maize microbiome. Microbiome.

[41] Agarwal A., Agashe D. (2020). The red flour beetle Tribolium castaneum: A model for host-microbiome interactions. PLoS ONE.

[42] Berasategui A., Salem H. (2020). Microbial determinants of folivory in insects. Cellular Dialogues in the Holobiont.

[43] Aloni R., Aloni R. (2021). How vascular differentiation in hosts is regulated by parasitic plants and gall-inducing insects. Vascular Differentiation and Plant Hormones.

[44] Gloder G., Bourne M.E., Verreth C., Wilberts L., Bossaert S., Crauwels S., Dicke M., Poelman E.H., Jacquemyn H., Lievens B. (2021). Parasitism by endoparasitoid wasps alters the internal but not the external microbiome in host caterpillars. Anim. Microbiome.

[45] Yang X., Hui Y., Zhu D., Zeng Y., Zhao L., Yang X., Wang Y. (2022). The diversity of bacteria associated with the invasive gall wasp *Dryocosmus kuriphilus*, its galls and a specialist parasitoid on chestnuts. Insects.

[46] Wemheuer F., Taylor J.A., Daniel R., Johnston E., Meinicke P., Thomas T., Wemheuer B. (2020). Tax4Fun2: Prediction of habitat-specific functional profiles and functional redundancy based on 16S rRNA gene sequences. Environ. Microbiome.

[47] Sansupa C., Wahdan S.F.M., Hossen S., Disayathanoowat T., Wubet T., Purahong W. (2021). Can we use functional annotation of prokaryotic taxa (FAPROTAX) to assign the ecological functions of soil bacteria?. Appl. Sci..

[48] Dong Y., Zhang Z.-R., Mishra S., Wong A.C.-N., Huang J.-F., Wang B., Peng Y.-Q., Gao J. (2022). Diversity and metabolic potentials of microbial communities associated with pollinator and cheater fig wasps in fig-fig wasp mutualism system. Front. Microbiol..

[49] Bálint M., Márton O., Schatz M., Düring R.A., Grossart H.P. (2018). Proper experimental design requires randomization/balancing of molecular ecology experiments. Ecol. Evol..

[50] De Cock M., Virgilio M., Vandamme P., Augustinos A., Bourtzis K., Willems A., De Meyer M. (2019). Impact of sample preservation and manipulation on insect gut microbiome profiling. A test case with fruit flies (Diptera, Tephritidae). Front. Microbiol..

[51] Hu Z., Zhang Z., Zhou Y., Zhao H., Li Y., Luo Y. (2024). Characterization of larval gut microbiota of two endoparasitoid wasps associated with their common host, *Plutella xylostella* (Linnaeus) (Lepidoptera: Plutellidae). Microbiol. Spectr..

[52] Gómez-Govea M.A., Peña-Carillo K.I., Ruiz-Ayma G., Guzmán-Velasco A., Flores A.E., Ramírez-Ahuja M., Rodríguez-Sánchez I. (2024). Unveiling the microbiome diversity in *Telenomus* (Hymenoptera: Scelionidae) parasitoid wasps. Insects.

[53] Brucker R.M., Bordenstein S.R. (2013). The hologenomic basis of speciation: Gut bacteria cause hybrid lethality in the genus *Nasonia*. Science.

[54] Hammer T.J., Dickerson J.C., Fierer N. (2015). Evidence-based recommendations on storing and handling specimens for analyses of insect microbiota. PeerJ.

[55] Caporaso J.G., Lauber C.L., Walters W.A., Berg-Lyons D., Huntley J., Fierer N., Owens S.M., Betley J., Fraser L., Bauer M. (2012). Ultra-high-throughput microbial community analysis on the Illumina HiSeq and MiSeq platforms. ISME J..

[56] Walters W., Hyde E.R., Berg-Lyons D., Ackermann G., Humphrey G., Parada A., Gilbert J.A., Jansson J.K., Caporaso J.G., Fuhrman J.A. (2016). Improved bacterial 16S rRNA gene (V4 and V4-5) and fungal internal transcribed spacer marker gene primers for microbial community surveys. mSystems.

[57] Toju H., Tanabe A.S., Yamamoto S., Sato H. (2012). High-coverage ITS primers for the DNA-based identification of ascomycetes and basidiomycetes in environmental samples. PLoS ONE.

[58] Klindworth A., Pruesse E., Schweer T., Peplies J., Quast C., Horn M., Glöckner F.O. (2013). Evaluation of general 16S ribosomal RNA gene PCR primers for classical and next-generation sequencing-based diversity studies. Nucleic Acids Res..

[59] Callahan B.J., McMurdie P.J., Rosen M.J., Han A.W., Johnson A.J.A., Holmes S.P. (2016). DADA2: High-resolution sample inference from Illumina amplicon data. Nat. Methods.

[60] Bolyen E., Rideout J.R., Dillon M.R., Bokulich N.A., Abnet C.C., Al-Ghalith G.A., Alexander H., Alm E.J., Arumugam M., Asnicar F. (2019). Reproducible, interactive, scalable and extensible microbiome data science using QIIME 2. Nat. Biotechnol..

[61] Quast C., Pruesse E., Yilmaz P., Gerken J., Schweer T., Yarza P., Peplies J., Glöckner F.O. (2013). The SILVA ribosomal RNA gene database project: Improved data processing and web-based tools. Nucleic Acids Res..

[62] Katoh K., Standley D.M. (2013). MAFFT multiple sequence alignment software version 7: Improvements in performance and usability. Mol. Biol. Evol..

[63] Lozupone C., Knight R. (2005). UniFrac: A new phylogenetic method for comparing microbial communities. Appl. Environ. Microbiol..

[64] Caporaso J.G., Kuczynski J., Stombaugh J., Bittinger K., Bushman F.D., Costello E.K., Fierer N., Peña A.G., Goodrich J.K., Gordon J.I. (2010). QIIME allows analysis of high-throughput community sequencing data. Nat. Methods.

[65] Lozupone C., Knight R. (2008). Species divergence and the measurement of microbial diversity. FEMS Microbiol. Rev..

[66] Oksanen J., Blanchet F.G., Friendly M., Kindt R., Legendre P., McGlinn D., Minchin P.R., O’Hara R.B., Simpson G.L., Solymos P. (2024). Vegan: Community Ecology Package; R Package.

[67] Douglas G.M., Maffei V.J., Zaneveld J.R., Yurgel S.N., Brown J.R., Taylor C.M., Huttenhower C., Langille M.G.I. (2020). PICRUSt2 for prediction of metagenome functions. Nat. Biotechnol..

[68] Louca S., Parfrey L.W., Doebeli M. (2016). Decoupling function and taxonomy in the global ocean microbiome. Science.

[69] Nguyen N.H., Song Z., Bates S.T., Branco S., Tedersoo L., Menke J., Schilling J.S., Kennedy P.G. (2016). FUNGuild: An open annotation tool for parsing fungal community datasets by ecological guild. Fungal Ecol..

[70] R Core Team (2023). R: A Language and Environment for Statistical Computing.

[71] McMurdie P.J., Holmes S. (2013). phyloseq: An R package for reproducible interactive analysis and graphics of microbiome census data. PLoS ONE.

[72] Csárdi G., Nepusz T. (2006). The igraph software package for complex network research. InterJournal Complex Syst..

[73] Douglas A.E. (2019). Simple animal models for microbiome research. Nat. Rev. Microbiol..

[74] Brucker R.M., Bordenstein S.R. (2012). Speciation by symbiosis. Trends Ecol. Evol..

[75] Douglas A.E. (2014). The molecular basis of bacterial–insect symbiosis. J. Mol. Biol..

[76] Sugio A., Dubreuil G., Giron D., Simon J.C. (2015). Plant-insect interactions under bacterial influence: Ecological implications and underlying mechanisms. J. Exp. Bot..

[77] Ley R.E., Hamady M., Lozupone C., Turnbaugh P.J., Ramey R.R., Bircher J.S., Schlegel M.L., Tucker T.A., Schrenzel M.D., Knight R. (2008). Evolution of mammals and their gut microbes. Science.

[78] Dittmer J., Brucker R.M. (2021). When your host shuts down: Larval diapause impacts host-microbiome interactions in *Nasonia vitripennis*. Microbiome.

[79] Kwong W.K., Moran N.A. (2016). Gut microbial communities of social bees. Nat. Rev. Microbiol..

[80] Wang G.H., Zheng R., Wang Q., Wu R., Paradkar P.N., Hoffmann A.A. (2023). Holobiont perspectives on tripartite interactions among microbiota, mosquitoes, and pathogens. ISME J..

[81] Bordenstein S.R., Theis K.R. (2015). Host biology in light of the microbiome: Ten principles of holobionts and hologenomes. PLoS Biol..

[82] Gätjens-Boniche O., Jiménez-Madrigal J.P., Whetten R.W., Valenzuela-Diaz S., Alemán-Gutiérrez A., Hanson P.E., Pinto-Tomás A.A. (2023). Microbiome and plant cell transformation trigger insect gall induction in cassava. Front. Plant Sci..

[83] Roy S., Mukherjee A., Gautam A., Bera D., Das A. (2022). Chemical arms race: Occurrence of chemical defense and growth regulatory phytochemical gradients in insect-induced foliar galls. Proc. Natl. Acad. Sci. India B Biol. Sci..

[84] Hammer T.J., De Clerck-Floate R., Tooker J.F., Price P.W., Miller D.G., Connor E.F. (2021). Are bacterial symbionts associated with gall induction in insects?. Arthropod Plant Interact..

