# Supplementary material for: Niche Differentiation and Predicted Functions of Microbiomes in a Tri-Trophic Willow–Gall (Euura viminalis)–Parasitoid Wasp System
Source: Insects. 2025 Dec 29;17(1):43. doi: 10.3390/insects17010043 (PMC12842123; doi:10.3390/insects17010043)
Supplement: Supplementary file 1 [file insects-17-00043-s001.zip › insects-4014483-supplementary.pdf]

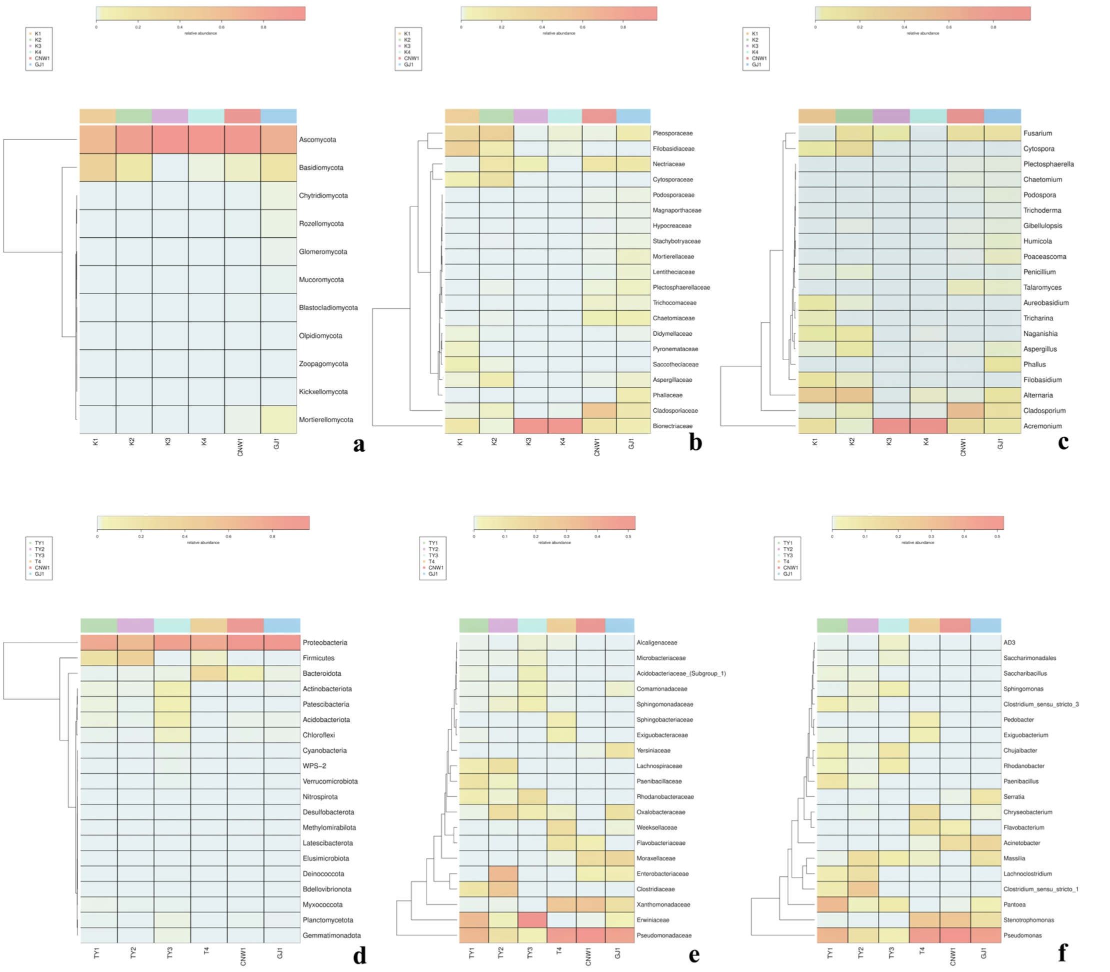

Figure S1. Taxonomic heatmaps across niches (ITS: a–c; 16S: d–f). (a,d) Phylum; (b,e) Family; (c,f) Genus; Colors show row-scaled relative abundance with hierarchical clustering of taxa.

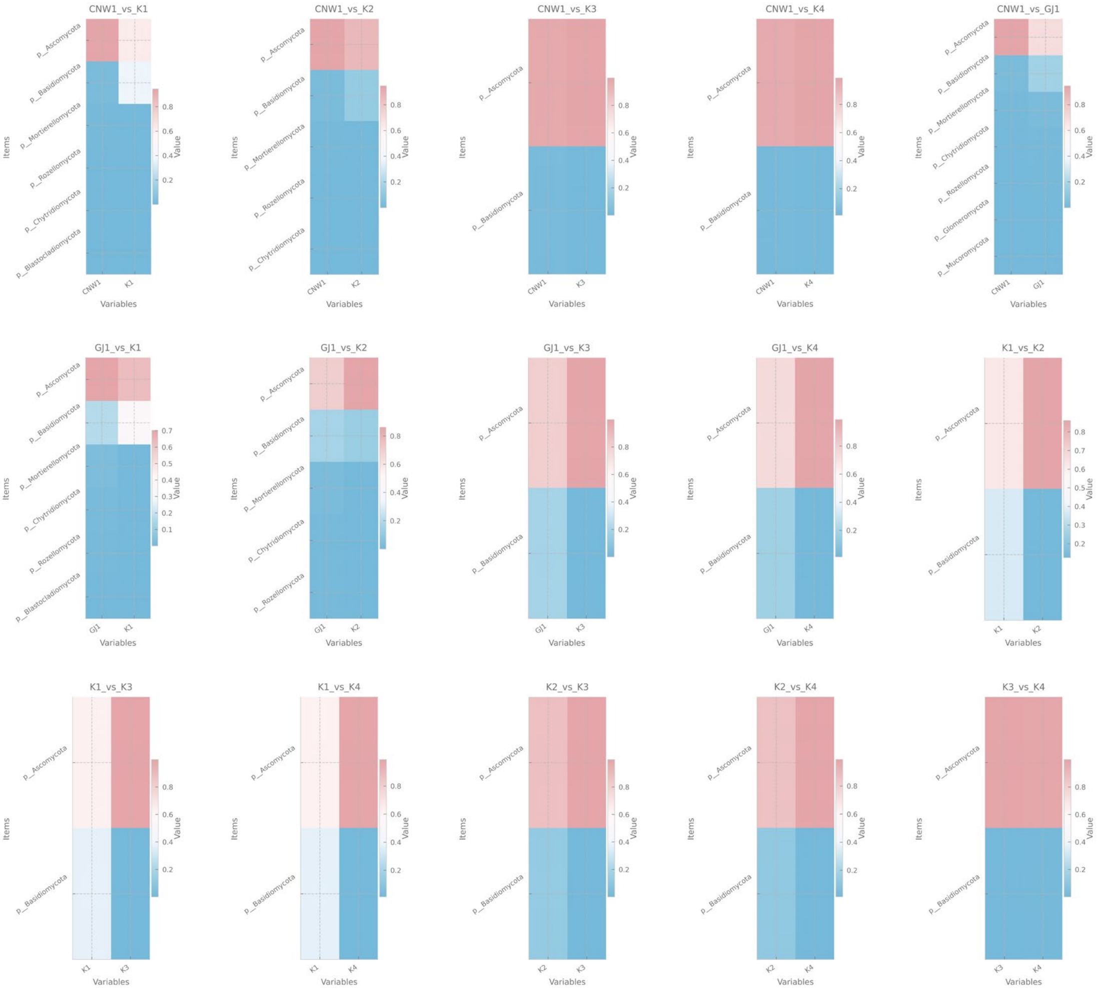

Figure S2. Pairwise differential-abundance heatmaps for fungal phyla (ITS). Each mini-panel is one contrast; rows are genera. Color shows direction (red = higher in the first group, blue = higher in the second), with intensity proportional to effect/q (BH-FDR).

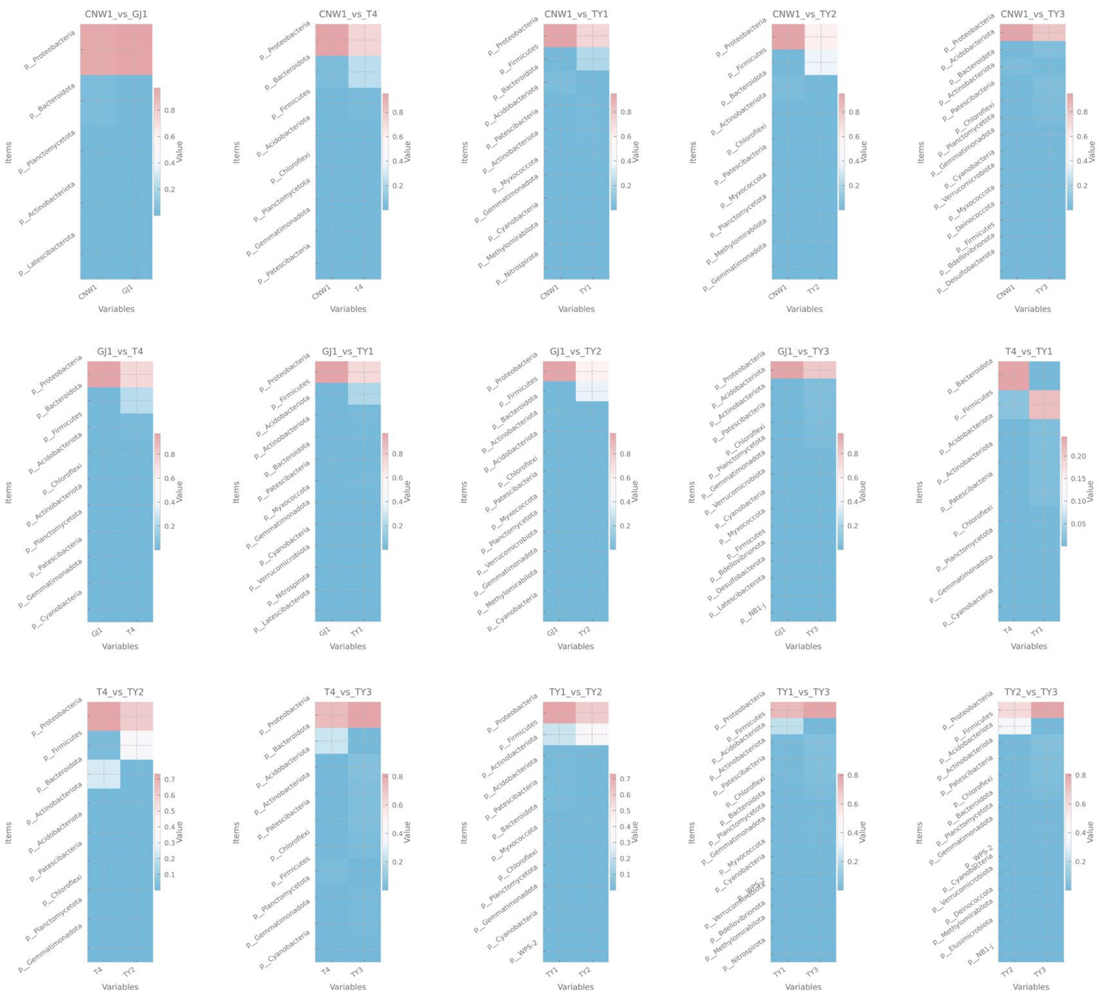

Figure S3. Pairwise differential-abundance heatmaps for fungal phyla (16S).

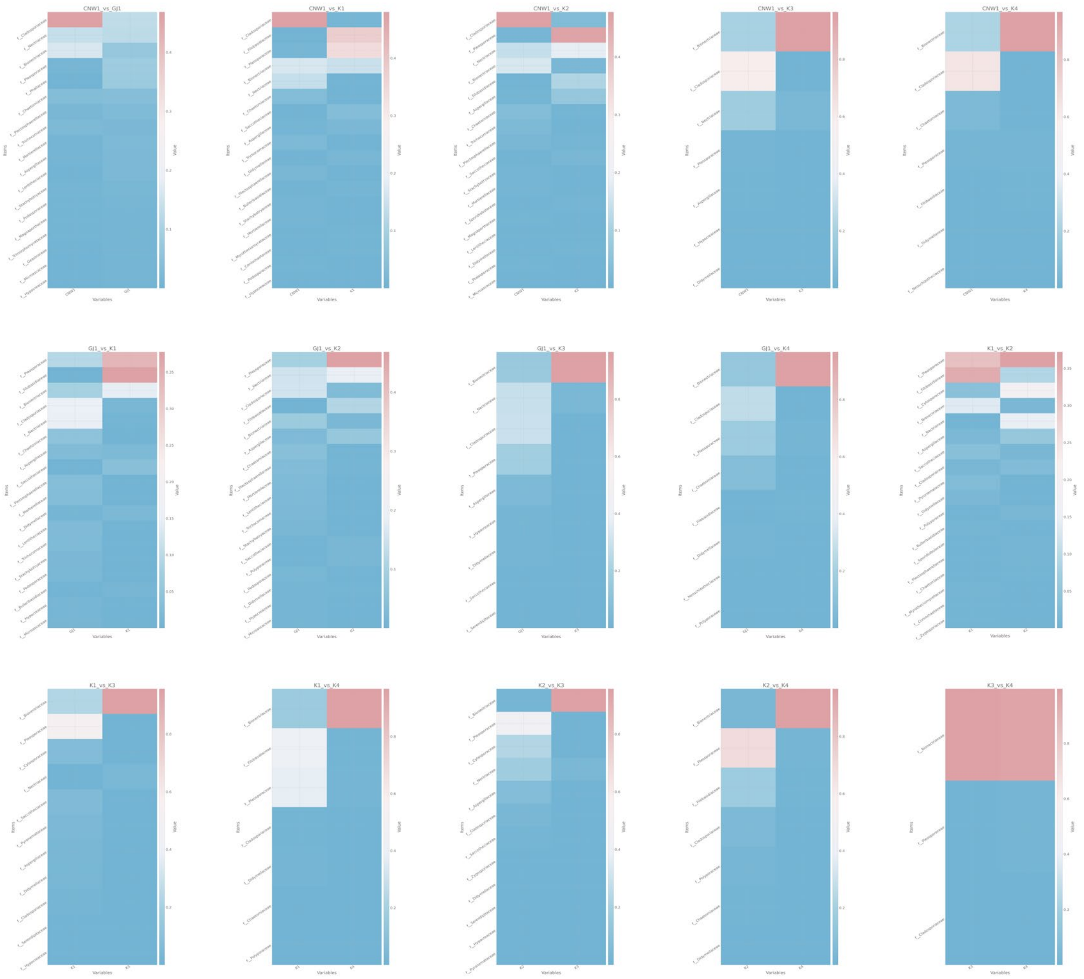

Figure S4. Pairwise differential-abundance heatmaps for family phyla (ITS).

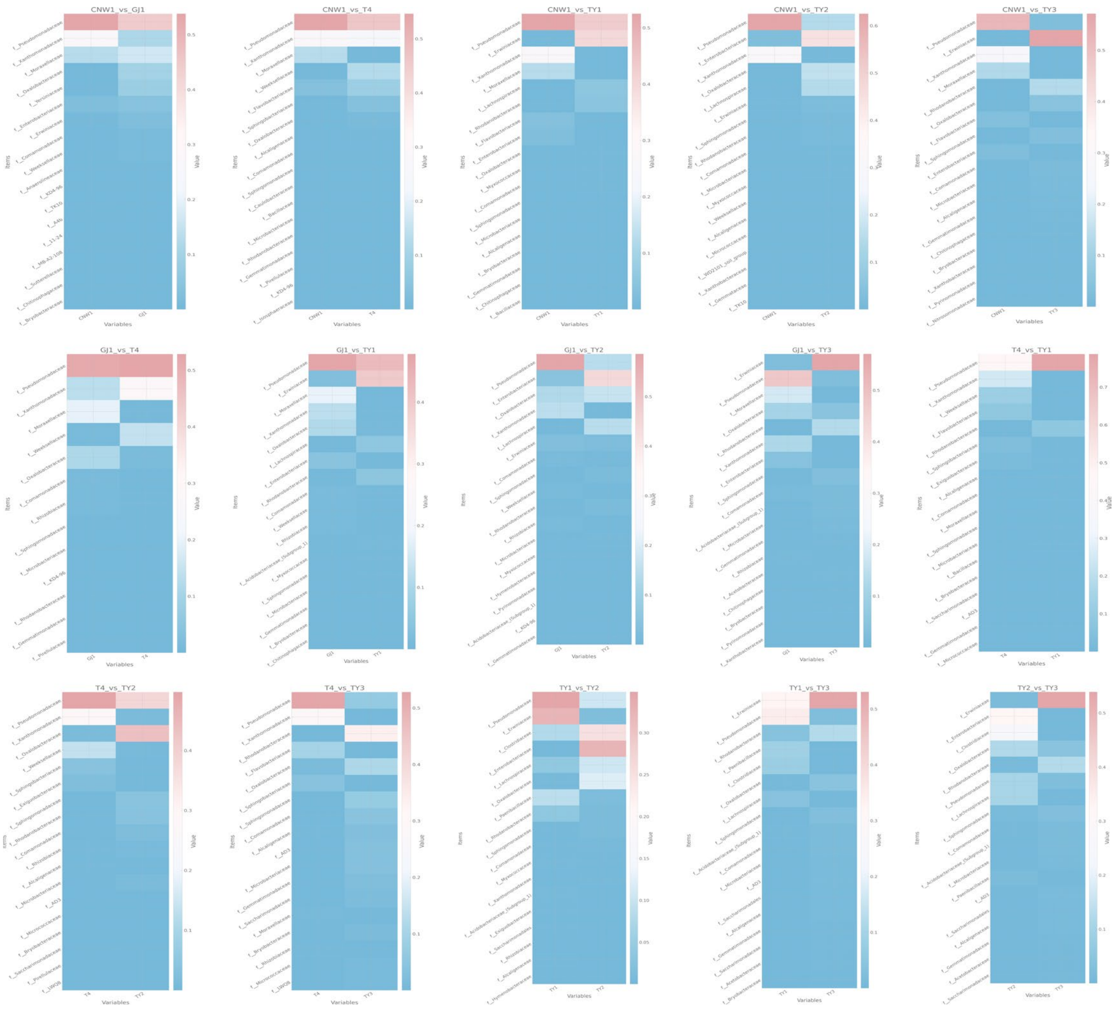

Figure S5. Pairwise differential-abundance heatmaps for family phyla (16S).

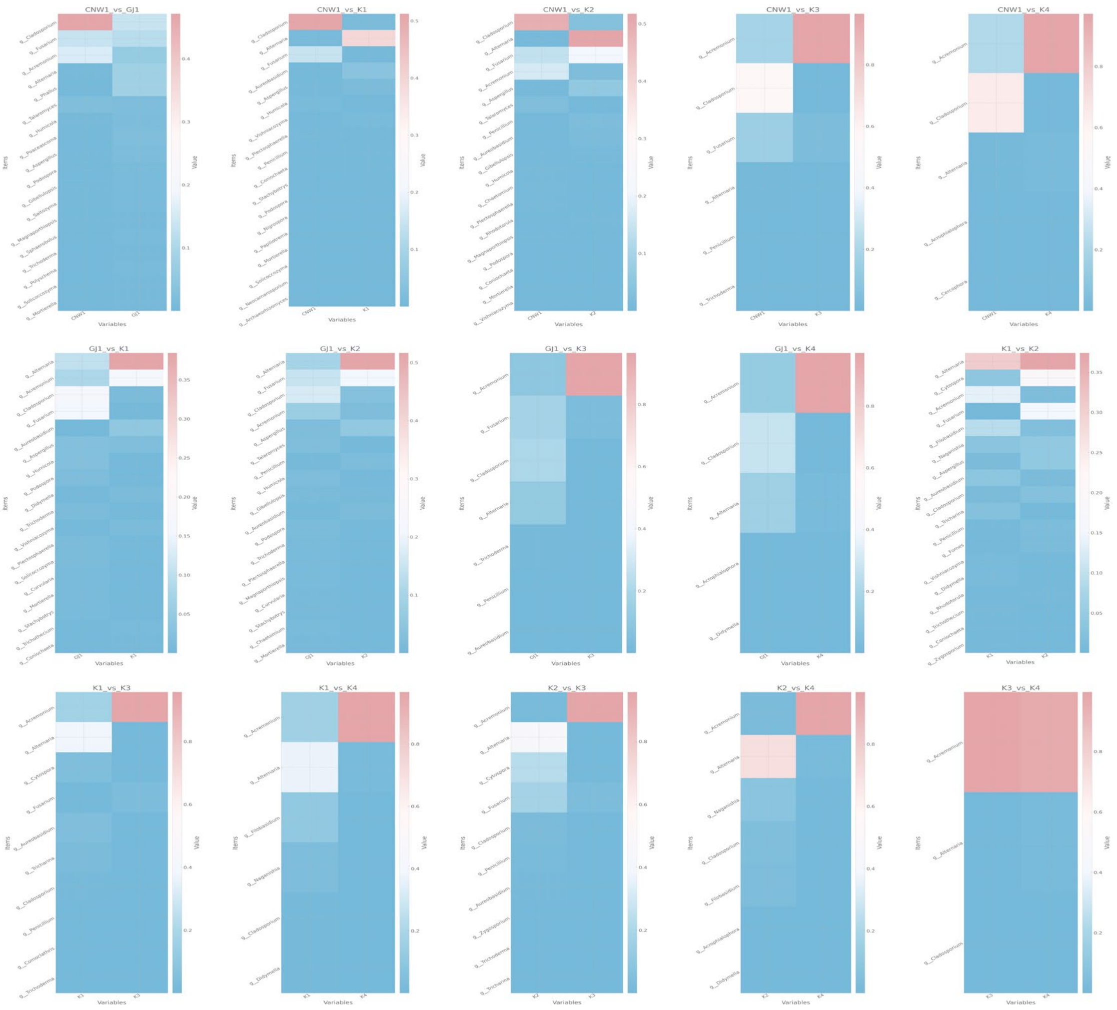

Figure S6. Pairwise differential-abundance heatmaps for genera phyla (ITS).

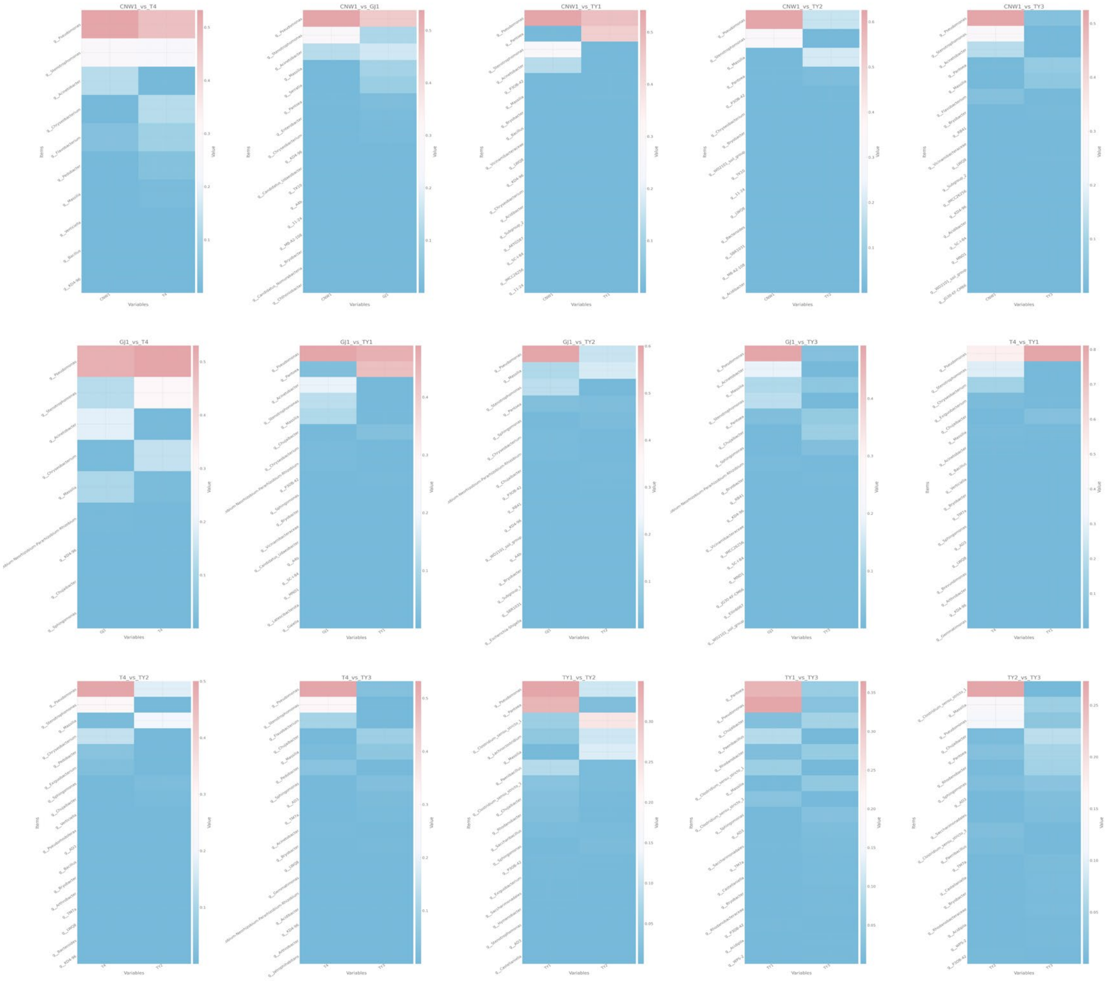

Figure S7. Pairwise differential-abundance heatmaps for genera phyla (16S).

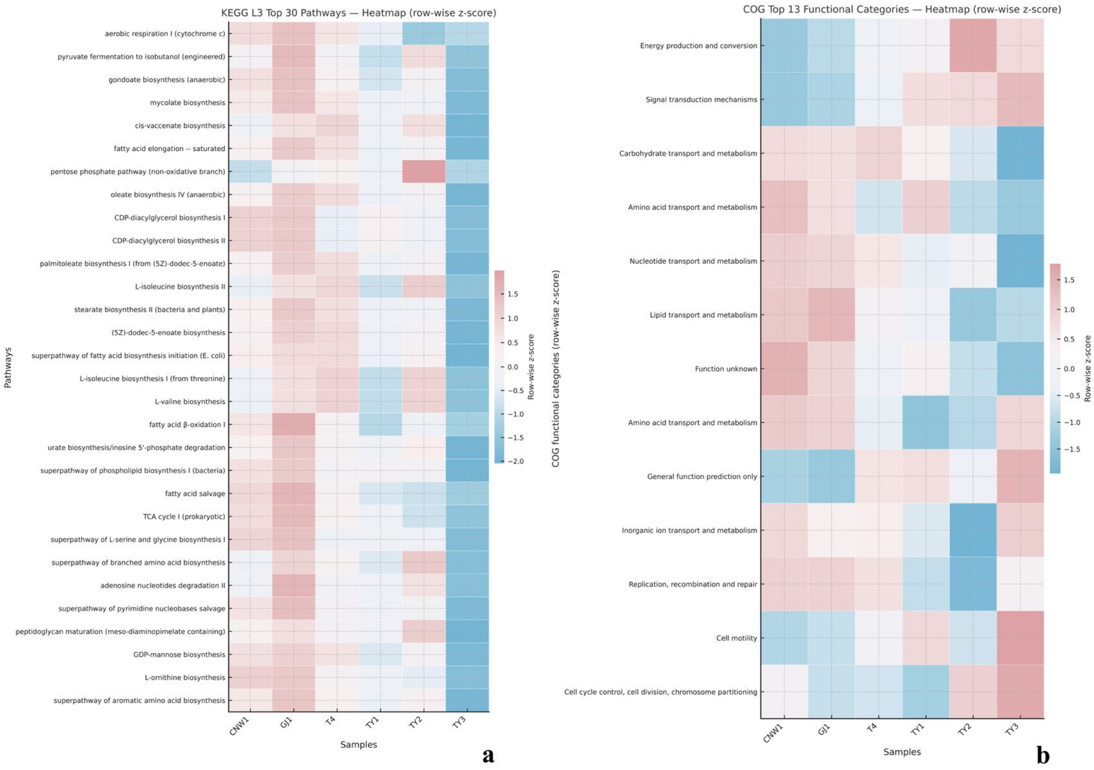

Figure S8. Functional prediction heatmaps (16S). (a) KEGG Level-3 top-30 pathways; (b) COG 13 functional categories. Values are row-wise z-scores across samples (CNW1, GJ1, T4, TY1, TY2, TY3); warmer colors indicate within-pathway enrichment and cooler colors indicate depletion.

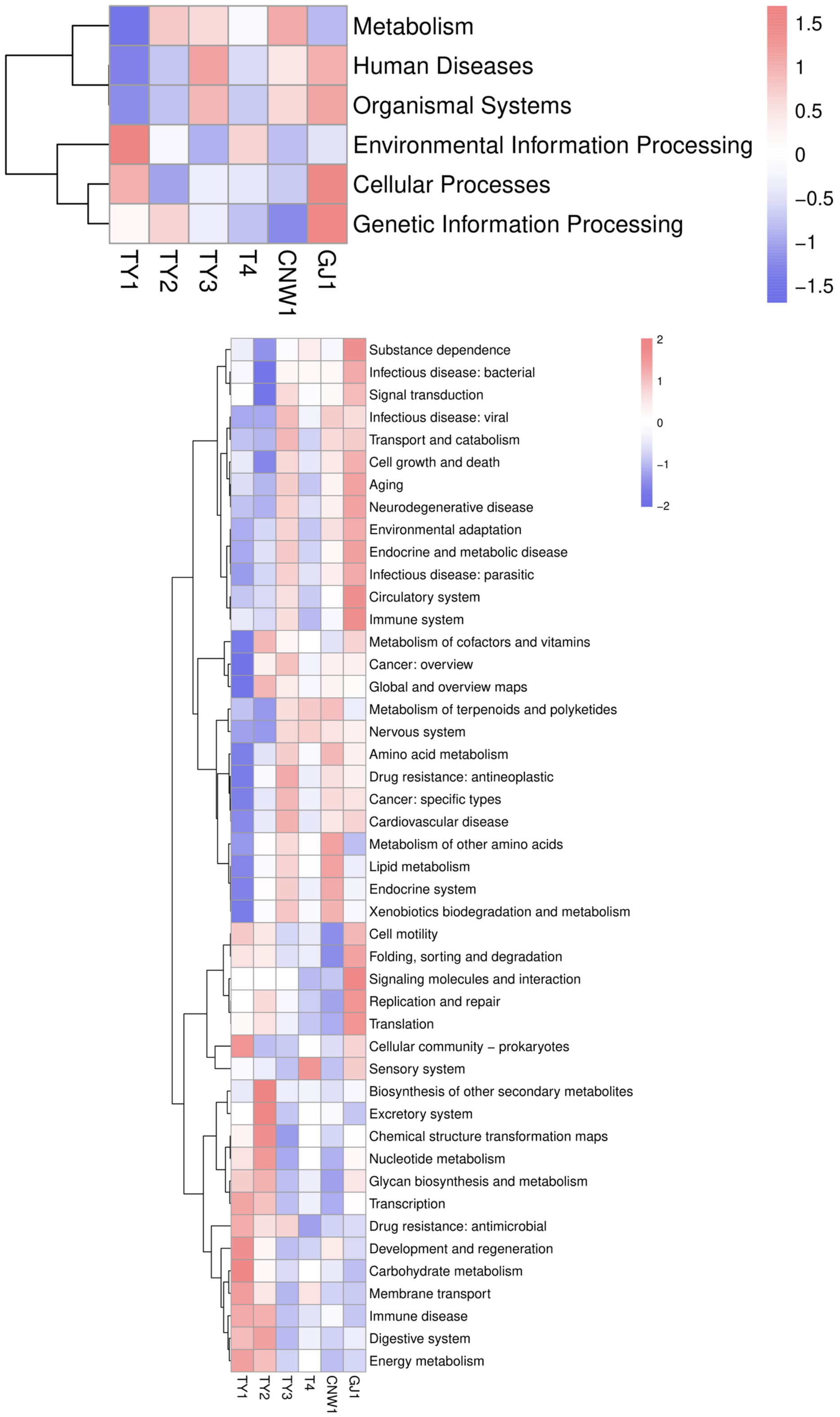

Figure S9. KEGG functional profiles (16S, PICRUSt2). (a) Heatmap of KEGG Level-1 categories; (b) heatmap of representative Level-2 pathways. Values are row-wise z-scores across samples (CNW1, GJ1, T4, TY1–TY3), with hierarchical clustering indicating sample similarity.
